# A computational framework for complex disease stratification from multiple large-scale datasets

**DOI:** 10.1186/s12918-018-0556-z

**Published:** 2018-05-29

**Authors:** Bertrand De Meulder, Diane Lefaudeux, Aruna T. Bansal, Alexander Mazein, Amphun Chaiboonchoe, Hassan Ahmed, Irina Balaur, Mansoor Saqi, Johann Pellet, Stéphane Ballereau, Nathanaël Lemonnier, Kai Sun, Ioannis Pandis, Xian Yang, Manohara Batuwitage, Kosmas Kretsos, Jonathan van Eyll, Alun Bedding, Timothy Davison, Paul Dodson, Christopher Larminie, Anthony Postle, Julie Corfield, Ratko Djukanovic, Kian Fan Chung, Ian M. Adcock, Yi-Ke Guo, Peter J. Sterk, Alexander Manta, Anthony Rowe, Frédéric Baribaud, Charles Auffray, the U-BIOPRED Study Group and the eTRIKS Consortium

**Affiliations:** 1 European Institute for Systems Biology and Medicine, CNRS-ENS-UCBL, EISBM, 50 Avenue Tony Garnier, 69007 Lyon, France; 2 Acclarogen Ltd, St John’s Innovation Centre, Cambridge, CB4 OWS UK; 3 Data Science Institute, Imperial College, London, SW7 2AZ UK; 4 Janssen Research and Development Ltd, High Wycombe, HP12 4DP UK; 5 UCB Pharma S.A, 1420 Braine-l’Alleud, Belgium; 6 UCB Celltech, 208 Bath Road, Slough, SL13WE UK; 7 Roche Ltd, Welwyn Garden City, AL7 1TW UK; 8 AstraZeneca Ltd, Alderley Park, Macclesfield, SK10 4TG UK; 9 Target Sciences, GlaxoSmithKline, Gunnels Wood Road, Stevenage, SG1 2NY UK; 10 Faculty of Medicine, University of Southampton, Southampton, SO17 1BJ UK; 11 AstraZeneca R & D, 43150 Mölndal, Sweden; 12 Arateva R & D Ltd, Nottingham, NG1 1GF UK; 13 National Hearth and Lung Institute, Imperial College London, London, SW3 6LY UK; 14 Department of Respiratory Medicine, Academic Medical Centre, University of Amsterdam, Amsterdam, AZ1105 The Netherlands; 15 Research Informatics, Roche Diagnostics GmbH, 82008 Unterhaching, Germany; 16 Janssen Research and Development Ltd, Spring House, PA 19002 USA

**Keywords:** Molecular signatures, ‘Omics data, Stratification, Systems medicine

## Abstract

**Background:**

Multilevel data integration is becoming a major area of research in systems biology. Within this area, multi-‘omics datasets on complex diseases are becoming more readily available and there is a need to set standards and good practices for integrated analysis of biological, clinical and environmental data. We present a framework to plan and generate single and multi-‘omics signatures of disease states.

**Methods:**

The framework is divided into four major steps: dataset subsetting, feature filtering, ‘omics-based clustering and biomarker identification.

**Results:**

We illustrate the usefulness of this framework by identifying potential patient clusters based on integrated multi-‘omics signatures in a publicly available ovarian cystadenocarcinoma dataset. The analysis generated a higher number of stable and clinically relevant clusters than previously reported, and enabled the generation of predictive models of patient outcomes.

**Conclusions:**

This framework will help health researchers plan and perform multi-‘omics big data analyses to generate hypotheses and make sense of their rich, diverse and ever growing datasets, to enable implementation of translational P4 medicine.

**Electronic supplementary material:**

The online version of this article (10.1186/s12918-018-0556-z) contains supplementary material, which is available to authorized users.

## Background

Since the early days of medicine, practitioners have always combined their observations from patient examinations with their medical knowledge and experience to diagnose medical conditions and find treatments tailored to the patient [1]. Nowadays, this rationale includes the integration of molecular, clinical, imaging information and other data sources to inform diagnosis and prognosis [2] or in other words, personalised medicine.

Various data integration methods developed through systems biology and computer science are now available to researchers. These methods aim at bridging the gap between the vast amounts of data generated in an ever-cheaper way [3] and our understanding of biology reflecting the complexity of biological systems [4]. Promises of data integration are the reduced cost of clinical trials, better statistical power, more accurate hypothesis generation and ultimately, individualised and cheaper healthcare [2].

However, a lack of communication exists between the fields of clinical medicine and systems biology, bioinformatics and biostatistics, as suggested by the reluctance or distrust to recent developments of personalised medicine by the medical community [1, 5, 6]. To address this issue, we developed a computational/analysis framework that aims at facilitating communication between healthcare professionals, computational biologists and bioinformaticians.

Among several ways of integrating data across biological levels, one of the components is multi-omics data integration. The identification of molecular signatures has been a focus of the biology and bioinformatics communities for over three decades. Early studies focused on a small number of molecules, paving the way for larger studies, eventually supporting the emergence of the ‘omics’ concept in the late 1990’s, starting with ‘genomics’ [7, 8]. Owing to both technical and biological advances, many classes of molecules have been studied by ‘omics technologies such as transcriptomics [9–11], proteomics [12, 13], lipidomics [14, 15], metabolomics (first mentioned in [16, 17]), the composition of the exhaled breath by breathomics (first mentioned in [18]) [19], and interactomics [20, 21], among others.

**Fig. 1:**
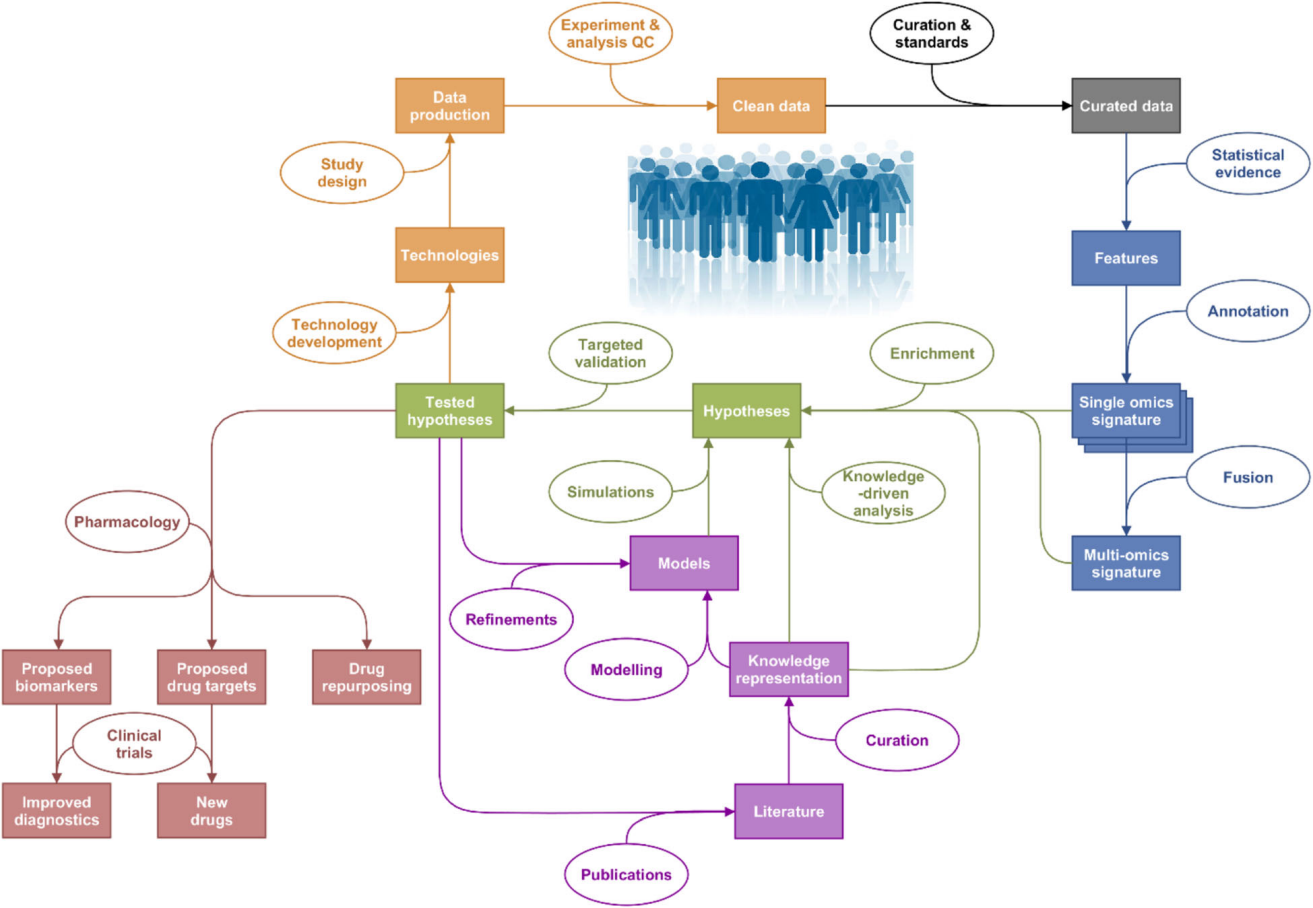
Outline of the Systems Medicine rationale. Represented in orange are the steps linked to quality data production, followed by curation in grey, identification of interesting features through statistical analysis in blue and hypothesis generation and their validation in green. Modelling and knowledge representation methods can inform the hypotheses generated through statistical analysis of generated hypotheses on their own (in purple). Outputs of this exercise are represented in red: drug repurposing, new drugs and improved diagnostics, with the help of clinical trials

Consequently, bioinformatics tools have been developed to analyse this new wealth of biological data, as reviewed in [22]. The concept of systems biology was developed first in the 1960’s [23, 24] to study biological organisms as complete and complex systems, integrating various sources of information (phenotypic data, molecular data, etc.) in combination with pathway/network analysis and mathematical modelling [25–33]. These systems approaches are highly suitable for the discovery of disease phenotypes (based on empirical recognition of observed characteristics) and so-called endotypes (capturing complex causative mechanisms in disease) [34]. The logical next step was to apply systems biology tools to improve clinical diagnosis, refine the endotypes leading to diseases, develop a comprehensive approach to the human body and assess an individual’s health in light of its ‘omics status. In this way the ‘systems medicine’ concept was born [35–41]. The systems medicine rationale is outlined in Fig. 1.

Any meaningful experiment relies on a robust, bias-controlled study design [42] using appropriate technologies, leading to the production of trustworthy quality-checked data. Data curation then aims at organising, annotating, integrating and preserving data from various sources for reuse and further integration. The next step is to identify relevant molecular features using statistical evidence. A tremendous and constantly growing number of methods is available for this purpose, making the process of method selection a crucial and challenging task. We provide some guidelines here but recommend that the reader turns to specialised reviews (such as [43]) for more insights on the relevance and appropriateness of individual methods. Once features are statistically selected, their annotation is required to interpret results and produce a single ‘omic signature. Annotation is a complex task that links identifiers from the technological platforms to existing entities (i.e. genes, peptides, metabolites, lipids, etc.) [44, 45]. If the data permit, information from several ‘omics platforms is integrated into multi-‘omics signatures. Single and multi ‘omics signatures ultimately serve to identify molecular mechanisms driving pathobiology.

Contextualisation of signatures with existing knowledge is now standard practice (e.g. ontology, enrichment and pathway analysis [46]), or performed with more advanced tools for data integration and visualisation such as a disease map [47]. Exploratory analysis using network-based information is valuable, with tools such as the STRING database [48], among many others. Hypotheses can then be formulated and tested in two ways, with external datasets and/or new experiments; or by modelling and knowledge representation (see review in [49] and disease maps examples in [47, 50–52]). With the help of systems pharmacology (see [53]), outcomes of this whole exercise are enabling: (i) identification of new potential drug targets associated with newly identified patient clusters, (ii) elucidation of potential biomarkers for diagnosis, (iii) repurposing of existing drugs and, ultimately, (iv) changes in diagnostic processes and development of new drugs and treatments for disease management. The key step in the systems medicine process is pattern recognition, for which a robust and step-wise framework is required.

### Definitions

Our article focuses on the identification of disease mechanisms through statistical analysis of raw data, annotation with up-to-date ontologies to generate *fingerprints* (biomarker signatures derived from data collected from a single technical platform), *handprints* (biomarker signatures derived from data collected within multiple technical platforms, either by fusion of multiple fingerprints or by direct integration of several data types) and interpretation on a pathway level to identify disease-driving mechanisms.

One way to better define the different endotypes is to generate molecular fingerprints (e.g. blood cell transcriptomics analysis yields genes differentially expressed between clinical populations [54]) and handprints (e.g. mRNA expression, DNA methylation and miRNA expression data fused to generate clusters of cancer patients [55]). The latter can be combined to study patients e.g. at the ‘blood biological compartment’ level, and linked with specific disease markers to better define the underlying biology, hence providing new avenues for therapy.

Despite the wealth of ‘omics analyses, little consensus exist on which statistical or bioinformatics methods to apply on each type of data set, nor on the ‘best’ integrative methods for their combined analysis (although standards exist for some data types, see [22]). Here, we present a generic framework to perform statistical and bioinformatics analyses of ‘omics measurements, starting from raw data management to multi-platform data integration, pathway and network modelling that has been adopted by the Innovative Medicines Initiative (IMI) U-BIOPRED Consortium (Unbiased BIOmarkers for the PREDiction of respiratory disease outcomes, http://www.ubiopred.eu) and extended in the eTRIKS Consortium (https://www.etriks.org/) to support a large number of national and European translational medicine projects. This article is not a review of the very large body of literature on relevant bioinformatics methods. Instead it describes generic steps in ‘omics data analysis to which many methods can be mapped to help multidisciplinary teams comprising clinical experts, wet-lab researchers, bioinformaticians, biostatisticians and computational systems biologists share a common understanding and communicate effectively throughout the systems medicine process [56].

We illustrate our pragmatic approach to the design and implementation of the analysis pipeline through a handprint analysis using the TCGA Research Network (The Cancer Genome Atlas – http://cancergenome.nih.gov/) Ovarian serous cystadenocarcinoma (OV) dataset.

### Data preparation: Quality control, correction for possible batch effects, missing data handling, and outlier detection

Quality Control (QC) comprises several important steps in data preparation. First, the platform-specific technical QC and normalisation are performed according to the standards of the respective fields of each particular technological platform.

Batch effects are a technical bias arising during study design and data production, due to variability in production platforms, staff, batches, reagent lots, etc. Their impact can be assessed using descriptive methods such as Principal Component Analysis (PCA) and graphical displays. Tools such as ComBat [57] and methodologies developed by van der Kloet [58] can be used to adjust for batch effects when necessary.

Missing data are features of all biological studies and arise for a variety of reasons. If the source of the missingness is unrelated to phenotype or biology, the missing data points can be classified as Missing Completely At Random (MCAR). Such missing values may be handled through imputation (to the mean, mode, mean of nearest neighbours, or by multiple imputation etc.) or by simple deletion [59].

Additional non-random missing data may arise due to assay- or platform-specific performances. For example, the measurement of abundances can fall below the lower limit of detection or quantitation (LLQ) of the instrument. In such instances, imputation is generally applied. Common methods include imputation to zero, LLQ, LLQ/2, or LLQ/√2; extrapolation and maximum likelihood estimation (MLE) can also be used [59].

**Fig. 2:**
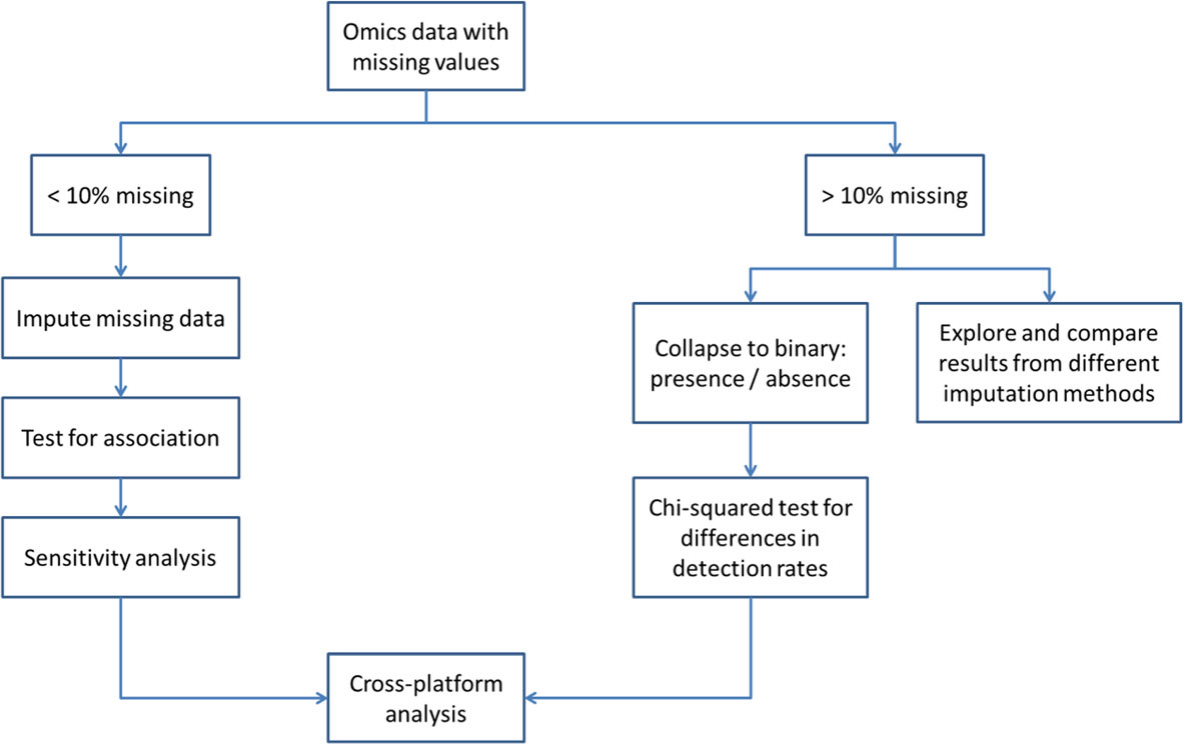
Process proposed for handling high levels of non-random missing data. If there are less than 10% missing values, data imputation is used, then tested for association (artificial associations might arise from the imputation process, which would then skew the analysis downstream) and submitted to a sensitivity analysis. If there are more than 10% missing values, we either collapse the feature/patient to a binary (presence/absence) scheme and run a χ^2^ test for difference in detection rates, or explore several imputation methods with highly cautious interpretation

Particular difficulty occurs in the analysis of mass spectrometry data, when it is impossible to distinguish MCAR data points from those below the LLQ of the technique. The combined levels of missing data often far exceed 10%. For these, the process depicted in the Fig. 2 is proposed.

Critical appraisal of the pattern of missingness is crucial. Where extensive imputation is applied, the robustness of imputation needs to be assessed by re-analysis, using a second imputation method, or by discarding the imputed values.

Outliers are expected in any biological/platform data. When these are clearly seen to arise due to technical artefacts (differences by many orders of magnitude, etc.), they should be discarded. Otherwise and in general, outlying values in biological data should be retained, flagged and subjected to statistical analysis.

When there is no community-wide consensus on a specific quality threshold for a particular biological data type, the research group generating the data applies quality filters on the basis of their knowledge and experience. Precise description of each data processing step should accompany each dataset to inform colleagues performing downstream analysis.

## Methods

### The framework concept

**Fig. 3:**
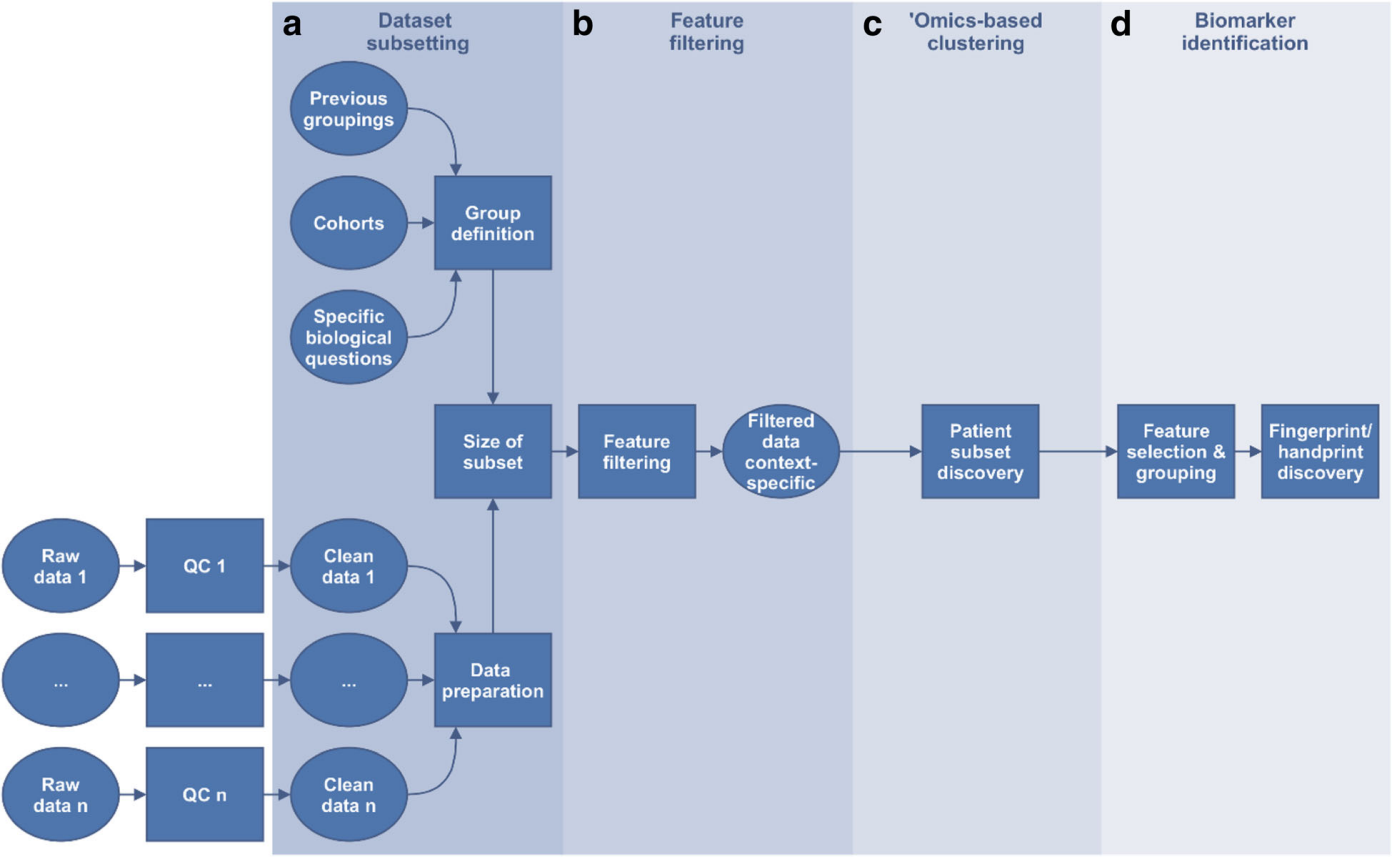
Overview of the framework. Starting from quality-checked and pre-processed ‘omics data, four key generic steps are highlighted: (**a**) dataset subsetting, including formulation of the biological question to be answered and data preparation, (**b**) feature filtering (optional step) where features that are uninformative in relation to the question can be removed, (**c**) ‘omics-based unsupervised clustering (optional step) aiming at finding groups of participants arising from the data structure using the (optionally filtered) features, and finally **d**) biomarker identification, including feature selection by bioinformatics means and machine learning algorithms for prediction

Several key generic steps in data analysis were identified and are highlighted in Fig. 3 below.

### Step 1: Dataset subsetting

This first box of Fig. 3 3 comprises two major steps: 1) formulating the biological question to be addressed and 2) preparing the data.

### Formulating the biological question

Several types of biological questions can be tackled, leading to different partitions of the dataset(s) to study. A partitioning scheme may rely on cohort definitions based on current state of the art, a specific biological question (e.g. comparing highly atopic to non-atopic severe asthmatics), or clustering results, obtained with clinical variables alone, distinct specific ‘omic or multi-‘omics clustering, etc.

### Data preparation

Depending on the question formulated at the previous step, data are then subsetted when appropriate. Then, an additional outlier detection check, data transformation and normalisation step can be performed, with methods described above. In this step, the statistical power that the analyst can expect (or the effect size that can be expected to be discovered) can be investigated (for more details on the computation of statistical power in ‘omics data analysis, see [60]). A decision on whether to split the datasets into training and validation sets is also made at this point (see section 4, replication of findings).

### Step 2: Feature filtering

Given the complexity and large amount of clinical and ‘omics data in a complex dataset, the number of features measured is vastly superior to the number of replicates creating various statistical challenges, i.e.. the ‘curse of dimensionality’ [61, 62]. Feature filtering (Fig. 3b) is therefore often used to select a subset of features relevant to the biological question studied, remove noise from the dataset and reduce the computing power and time needed [63–65].

Features can be filtered according to specific criteria, based for example on nominal *p*-values arising from comparison between groups. Indeed, several methods exist to perform feature filtering, based on mean expression values, p-values, fold changes, correlation values [66, 67], information content measures [68, 69], network-based metrics (connectivity, centrality [70, 71]) or using a non-linear machine learning algorithm [72]. We redirect the reader to the following reviews for more details [33, 73–75]. As this step might introduce bias into the downstream analyses, it is not always applied.

### Step 3: ‘Omics-based clustering

Clustering analysis groups elements so that objects in the same group are more similar to each other than to those in other groups (Fig. 3c). All methods available rely on similarity or distance measures and a clustering algorithm [76–78]. The most classical clustering methods may be categorized as ‘partitioning’ (constructing *k* clusters) or ‘hierarchical’ (seeking to build a hierarchy of clusters), and either agglomerative (each observation starts in its own cluster, and pairs of clusters are merged as one moves up the hierarchy, ending in a single cluster) or divisive (all observations start in the same cluster and splits are performed recursively as one moves down the hierarchy, ending with clusters containing one single observation).

It is important to note that clustering techniques are descriptive in nature and will yield clusters, whether they represent reality or not [76]. One way of finding out whether clusters represent reality is to assess their stability, with the consensus clustering approach [79] for example. Using different stable clustering algorithms on the same dataset and comparing them with the meta-clustering rationale [80] is a further step to assess if clusters represent accurately and reproducibly the biological situation in the data.

When several ‘omics datasets on the same patients are available, a handprint analysis can be performed with the Similarity Network Fusion (SNF) method to derive a patient-wise multi-‘omics similarity matrix [55]. Other methods for data integration in the context of subtype discovery are available such as iCluster [81], Multiple Dataset Integration [82], or Patient-Specific Data Fusion [83], further discussed in [84] or under development, for example by the European Stategra FP7 project (http://www.stategra.eu).

### Step 4: Biomarker identification

Steps 1 to 3 aim at finding groups of patients to best describe the biological condition(s), with respect to the questions addressed. Step 4 aims at 1) finding the smallest set of molecular features whose difference in abundance between these patient groups (Fig. 3d) enable their distinction (biomarkers) and 2) building classification models through machine-learning techniques, some of which use both feature reduction and classification model building together. The outcome is a fingerprint or handprint, depending on the number of different ‘omics datasets included in the analysis.

### Over-fitting and false-discovery rate control

As already mentioned, ‘omics technologies suffer from what is known as the ‘curse of dimensionality’, typically due to the large number of features (p) and low number of samples (n). As statistical methods were historically developed for a situation where the dimensions were n >> > p instead of the p >> > n situation, methods adjustments had to be made. The main issue in statistical analysis is the high type I error rate (false positives) in null hypothesis testing. Several ways of correcting for this have been developed, the most well-known and used being the Bonferroni correction and the Benjamini-Hochberg False Discovery Rate (FDR) controlling procedure [85]. Discussions are still ongoing in the statistics community as to which method is best to control the false positive rates in the context of ‘omics data analysis [46, 86, 87]. We therefore advise to split the data in testing and validation groups. Tests made within each group are corrected for FDR with the Benjamini-Hochberg’s procedure whenever possible or advised by domain experts, and only features detected in both groups should be considered for further analysis and interpretation.

Over-fitting may occur when a statistical model includes too many parameters relative to the number of observations. The over-fitted model describes random error instead of the underlying relationship of interest and performs poorly with independent data. In deriving prediction models therefore, a guiding principle is that there should be at least ten observations (or events) per predictor element [88] while simple models with few parameters should be favoured whenever possible.

All in all, the combination of internal replication, FDR correction and conservative over-fitting considerations allows the detection of interesting ‘omics features with a reference statistical foundation.

### Replication of findings

When a large number of statistical tests have been planned, a comprehensive adjustment for multiple testing can be detrimental to statistical power. Validation and replication of findings is therefore essential in order to avoid the widespread unvalidated biomarker syndrome that has plagued the vast majority of claimed biomarkers. Indeed, fewer than 1/1000 have proved clinically useful and approved by regulatory authorities [89–94]. For each combination of platform and sample type, an assessment can be made as to whether the data should be split into training and validation sets, or instead analysed as a single pool.

The predictive value of a biomarker identified after proper internal replication applies to the dataset in which it was discovered. Replication of findings in additional sample sets is a crucial step in producing clinically usable biomarkers and predictive models [95, 96] and should thus always be sought.

Once the feature filtering step is performed, the next step is to make sense of the results, either in a biological or mathematical manner. Biological annotation can be performed using pathways (see review in [97]) or functional categories (reviewed in [98]); however, this kind of analysis is hampered by factors such as statistical considerations (which method to use, independence between genes and between pathways, how to take into account the magnitude of the changes) and pathway architecture considerations (pathways can cross and overlap, meaning that if one pathway is truly affected, one may observe other pathways being significantly affected due to the set of overlapping genes and proteins involved) [99]. One way of overcoming those limitations is to use the complete genome-scale network of protein-protein interactions to define affected sub-regions of the network, with available academic [100, 101] and commercial solutions (e.g. MetaCore™ Thomson Reuters, IPA Ingenuity Pathway Analysis). A recent proposed solution is the disease map concept, following the examples of the Parkinson’s disease map [47], the Atlas of Cancer Signalling Networks [50] and the AlzPathway [51, 52] where an exhaustive set of relevant interactions to a particular disease are represented in details as a single network, which can then be analysed biologically and mathematically, with the supervision of domain experts for coverage and specificity [102].

## Results

### Application to a public domain dataset: TCGA OV dataset for handprint analysis

The Cancer Genome Atlas (TCGA, http://cancergenome.nih.gov/) is a joint effort of the National Cancer Institute (NCI) and the National Human Genome Research Institute (NHGRI) in the USA. It aims to accelerate our understanding of the molecular basis of cancer through application of genome analysis technologies. Among other functionalities, TCGA offers a freely available database of multi-‘omics datasets (including clinical data, imaging, DNA, mRNA and miRNA sequencing, protein, gene exon and miRNA expression, DNA methylation and copy number variation (CNV)) for several cancer types, with patient numbers ranging from a few dozens to above a thousand.

As a use case, the ovarian cancer OV dataset was chosen, as it comprises several ‘omics measurements for a large group of patients; this dataset has already been well characterized in several publications but without a data fusion analysis, in contrast to the glioblastoma TCGA dataset, for example [55]. It comprises data from a total of 586 patients, along with several ‘omics datasets (such as SNP, Exome, methylation…), as shown in the Table 1**.** below. All data matrices were downloaded using the Broad Institute FireBrowse TCGA interface (http://firebrowse.org/?cohort=OV&download_dialog=true#); the results shown here are based upon data generated by the TCGA Research Network.

**Table 1:** This table shows the number of cases in each ‘omics platform available for the TCGA Ovarian Serous Cystadenocarcinoma dataset (source: https://gdc.cancer.gov/)

Ovarian serous cystadenocarcinoma	Total	Exome	SNP	Methylation	mRNA	miRNA	Clinical
Cases	586	536	579	584	574	582	584

### Data preparation

**Fig. 4:**
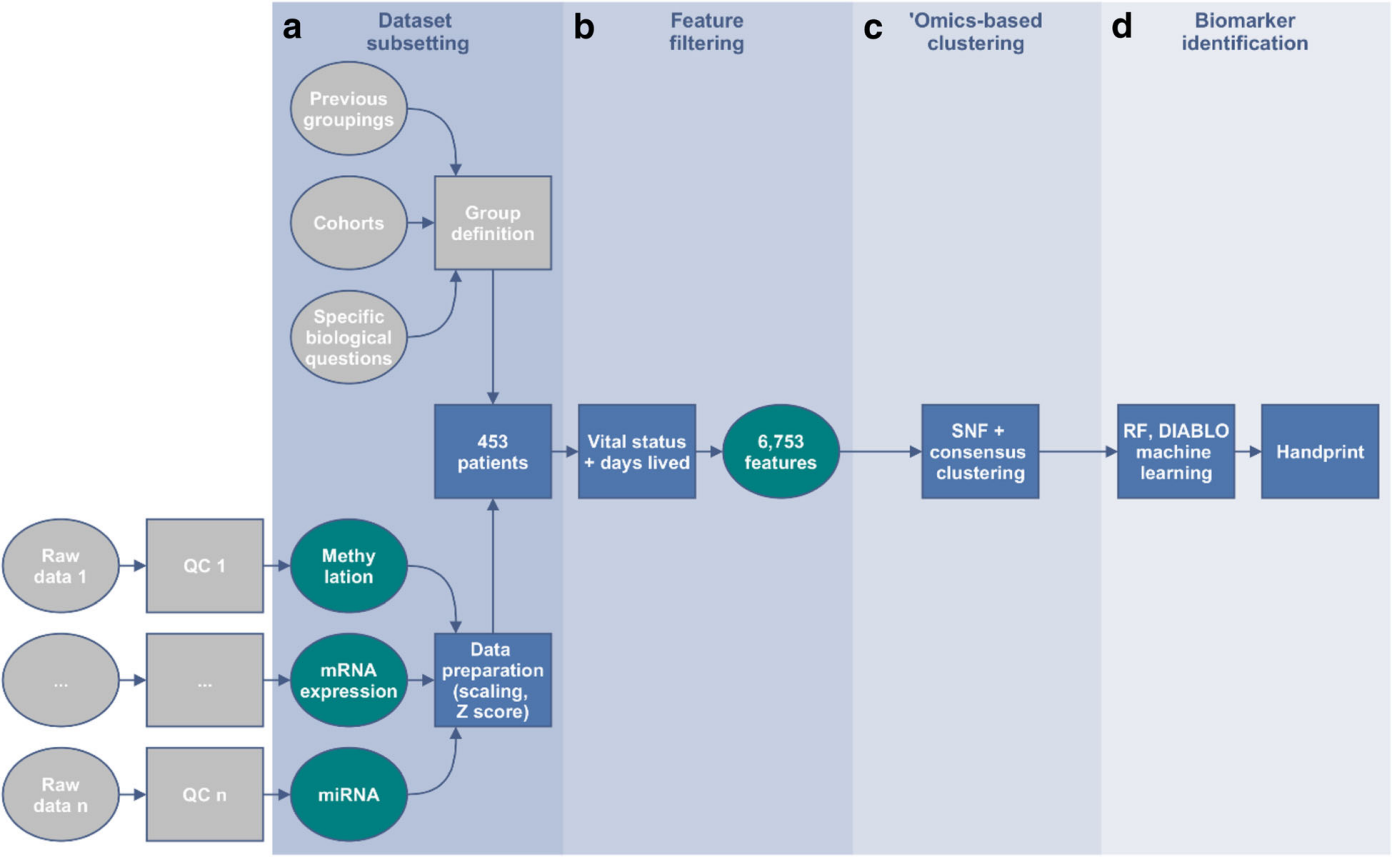
Framework outline for the TCGA handprint analysis with additional feature filtering. Each dataset was separately filtered based on nominal *p*-values < 0.05 when comparing alive versus deceased patients at the end of the study taking into account the total amount of days alive. A total of 6753 features were selected: 899 differentially methylated genes, 37 miRNAs and 5817 differentially expressed probesets. Consensus clustering on the fused similarity matrices determined the number of stable clusters that were viewed in a Kaplan-Meyer plot and tested for differential survival. Machine learning was then performed to identify candidate features predicting the identified groups: Recursive Feature Elimination (RFE) on a linear Support-Vector-Machine (SVM) model to identify informative features, followed by a Random Forest (RF) model building in parallel with DIABLO sPLS-DA on those features

We used the clinical, methylation, mRNA and miRNA data matrices from the 453 patients (out of a total of 586 patients) for which all four data types were available. The overview of the analysis is summarized in the Fig. 4.

### Feature selection

Preliminary analysis without feature selection was performed (data not shown). Briefly, this analysis led to the identification of four stable clusters, mainly differentiated by lymphatic and venous invasion status and clinical stage. Biologically speaking, the comparison of clusters led to the highlighting of well-known ovarian cancer biomarkers and pathways.

In order to produce a handprint more focused on the survival status of patients in the dataset, each ‘omics dataset was treated separately to identify features associated with survival status at the end of the study and overall survival time. The latter was obtained by summing the age (in days) of the participants at enrolment in the study and the post-study survival time, both values available in the clinical variables from the TCGA website. After data preparation including imputation of missing data in methylation and normalisation, linear models testing for survival status with survival time as a cofactor were fitted feature-wise and *p*-values for differential expression/abundance were derived. All features with a nominal p-value < 0.05 were selected. This yielded a total of 899 features in the methylation dataset, 37 miRNAs and 5817 probesets in transcriptomics.

### ‘Omics-based clustering

**Fig. 5:**
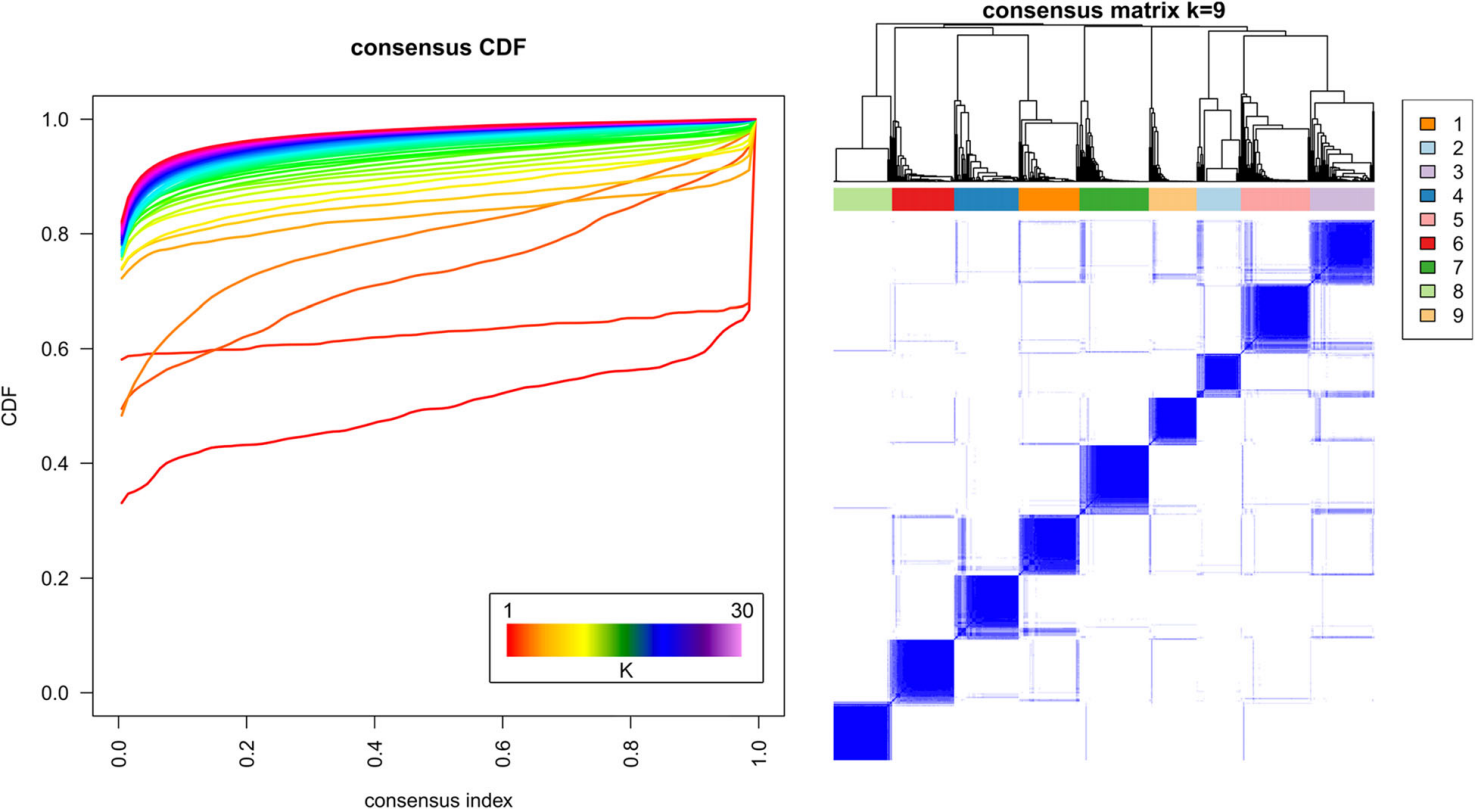
Consensus clustering results for the handprint analysis with feature filtering. A number of stable clustering schemes are available (k = 3, 6, 7, 8, 9). Nine clusters were chosen as the most informative, while keeping a low value of the deviation from ideal stability index and with clinical characteristics of the clusters statistically different in both survival time and survival status between clusters

Similarity matrices were derived from each filtered ‘omics dataset, which were fused with SNF, and spectral clustering with a consensus clustering step was applied to detect stable clusters, as shown in Fig. 5 below. The choice of the optimal number of stable clusters is based on two mathematical parameters: the deviation from ideal stability (DIS, a measure of the deviation from horizontality of the CDF curves in the left panel of the Fig. 5, the formulation of which can be found in the supplementary material of [103]), and the number of patients assigned in each cluster (clusters with fewer than 10 patients should be avoided [58]). The DIS across the number of clusters can be found in the Additional file 1. The DIS shows a minimal value for k = 3 clusters, but very similar values can be seen for k = 6, 7, 9, 10, 11 and 12. As it is clinically interesting to distinguish a higher number of clusters and to define clusters with different survival status, we chose the number of clusters associated with low DIS, no clusters with fewer than 10 patients, and statistically significant differences in survival status and survival time of patients, k = 9.

**Fig. 6:**
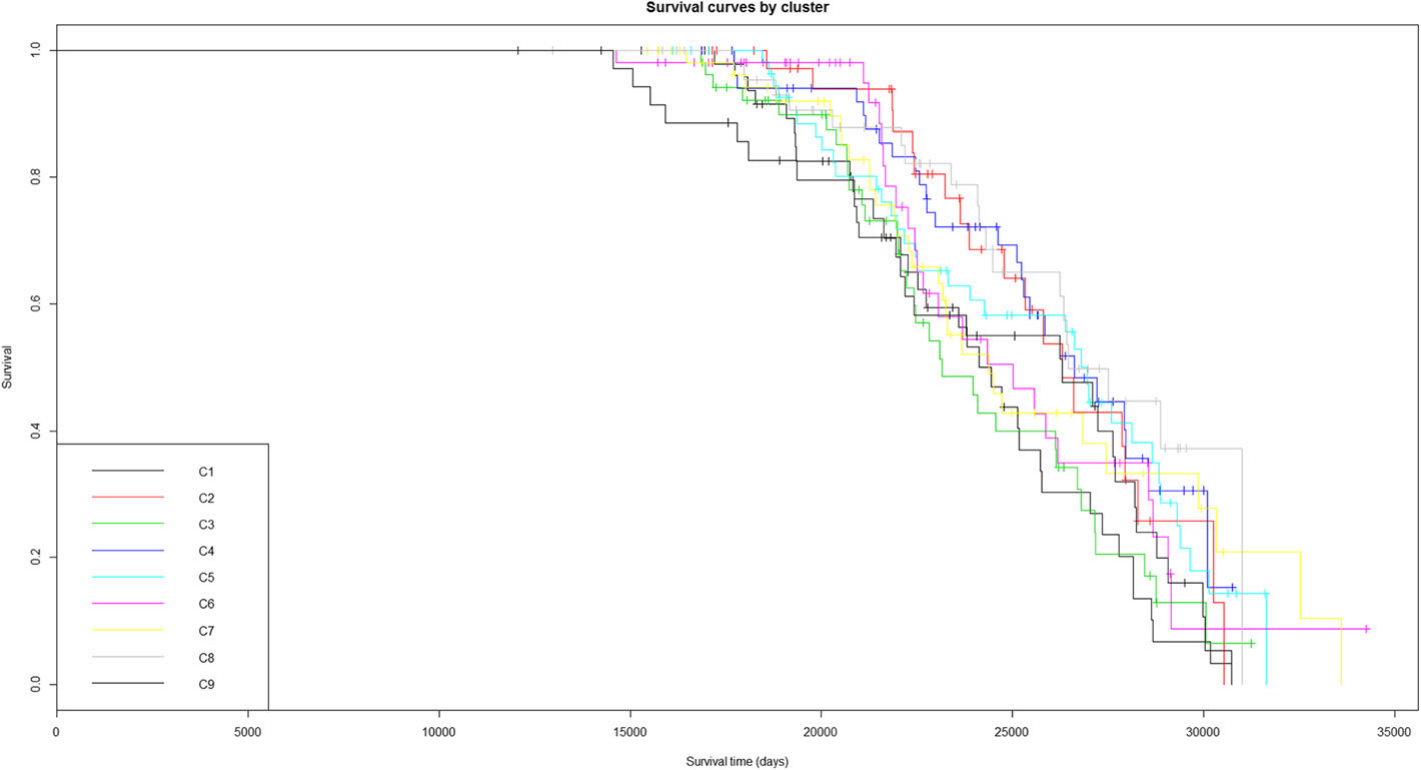
Kaplan-Meyer plot of survival for patients from the nine clusters revealed with the consensus clustering analysis. The x axis bears the total amount of days that patients have lived, i.e. the sum of their age at enrolment in the study plus the recorded amount of days they survived during the study, censored to the right by the end of measurements in the study (enrolment plus 4624 days)

The clinical characteristics of the nine clusters are shown in Table 2. Survival curves are also shown in the Kaplan-Meyer plot (Fig. 6). Survival status and survival time differ between the nine clusters, showing for example that patients in cluster 1 have a higher mortality rate.

**Table 2:** Clinical characteristics of the nine clusters found in the focused handprint analysis

Variables/clusters	C1 (*n* = 49)	C2 (n = 30)	C3 (*n* = 75)	C4 (*n* = 41)	C5 (*n* = 47)	C6 (*n* = 52)	C7 (*n* = 46)	C8 (*n* = 56)	C9 (*n* = 57)	*P*-value
Age at initial pathologic diagnosis (Yr)	57.6 ± 13.2	53.5 ± 8.16	59.8 ± 10.7	61.1 ± 12	60.2 ± 9.67	63.4 ± 11.8	59.8 ± 12.5	59.4 ± 11.6	60 ± 11.4	*3.40E-02* ^*2*^
Days from birth (Days)	−21,200 ± 4830	−19,700 ± 3030	−21,900 ± 3870	−22,700 ± 4260	−22,200 ± 2580	− 23,300 ± 4290	−22,000 ± 4560	−21,900 ± 4240	−22,200 ± 4140	*3.15E-02* ^*2*^
Days to death (Days (IQR))	1220 (725–1490)	1480 (1210–2360)	997 (404–1230)	949 (563–1360)	787 (512–1340)	1090 (680–1580)	978 (536–1450)	1070 (340–1440)	1290 (731–1700)	*2.11E-02* ^*1*^
Days to last followup (Days (IQR))	1090 (689–1460)	1200 (688–1550)	664 (238–1120)	763 (272–1820)	676 (185–1560)	804 (339–1560)	651 (347–1370)	816 (223–1370)	1280 (605–1690)	*3.74E-02* ^*1*^
Initial pathologic diagnosis method	Cytology: 9; Excisional biopsy: 2; Fine needle aspiration biopsy: 2; Incisional biopsy: 4; Tumor resection: 32	Cytology: 3; Excisional biopsy: 0; Fine needle aspiration biopsy: 0; Incisional biopsy: 0; Tumor resection: 27	Cytology: 12; Excisional biopsy: 0; Fine needle aspiration biopsy: 3; Incisional biopsy: 0; Tumor resection: 59; NA: 1	Cytology: 2; Excisional biopsy: 0; Fine needle aspiration biopsy: 2; Incisional biopsy: 1; Tumor resection: 36	Cytology: 9; Excisional biopsy: 2; Fine needle aspiration biopsy: 0; Incisional biopsy: 2; Tumor resection: 33; NA: 1	Cytology: 6; Excisional biopsy: 0; Fine needle aspiration biopsy: 1; Incisional biopsy: 0; Tumor resection: 44; NA: 1	Cytology: 2; Excisional biopsy: 0; Fine needle aspiration biopsy: 0; Incisional biopsy: 0; Tumor resection: 44	Cytology: 9; Excisional biopsy: 1; Fine needle aspiration biopsy: 0; Incisional biopsy: 3; Tumor resection: 43	Cytology: 5; Excisional biopsy: 0; Fine needle aspiration biopsy: 1; Incisional biopsy: 0; Tumor resection: 51	*3.28E-03* ^*3*^
Lymphatic invasion	No: 4; Yes: 9; NA: 36	No: 6; Yes: 10; NA: 14	No: 7; Yes: 19; NA: 49	No: 13; Yes: 5; NA: 23	No: 1; Yes: 17; NA: 29	No: 13; Yes: 6; NA: 33	No: 8; Yes: 21; NA: 17	No: 4; Yes: 8; NA: 44	No: 5; Yes: 14; NA: 38	*2.43E-02* ^*3*^
Neoplasm histologic grade	G1: 1; G2: 13; G3: 33; G4: 0; Gb: 1; Gx: 1	G1: 0; G2: 5; G3: 24; G4: 0; Gb: 0; Gx: 0; NA: 1	G1: 0; G2: 5; G3: 70; G4: 0; Gb: 0; Gx: 0	G1: 0; G2: 5; G3: 36; G4: 0; Gb: 0; Gx: 0	G1: 0; G2: 6; G3: 39; G4: 0; Gb: 0; Gx: 2	G1: 0; G2: 6; G3: 44; G4: 1; Gb: 0; Gx: 1	G1: 0; G2: 8; G3: 38; G4: 0; Gb: 0; Gx: 0	G1: 0; G2: 1; G3: 53; G4: 0; Gb: 0; Gx: 2	G1: 0; G2: 6; G3: 49; G4: 0; Gb: 0; Gx: 1; NA: 1	*1.89E-02* ^1^
Ethnicity	American Indian or Alaska native: 1; Asian: 1; Black or African American: 3; White: 43; NA: 1	American Indian or Alaska native: 0; Asian: 1; Black or African American: 2; White: 27; NA: 0	American Indian or Alaska native: 0; Asian: 3; Black or African American: 2; White: 68; NA: 2	American Indian or Alaska native: 0; Asian: 1; Black or African American: 3; White: 37; NA: 0	American Indian or Alaska native: 1; Asian: 1; Black or African American: 0; White: 41; NA: 4	American Indian or Alaska native: 0; Asian: 2; Black or African American: 4; White: 44; NA: 2	American Indian or Alaska native: 0; Asian: 3; Black or African American: 1; White: 41; NA: 1	American Indian or Alaska native: 0; Asian: 3; Black or African American: 2; White: 49; NA: 2	American Indian or Alaska native: 0; Asian: 0; Black or African American: 4; White: 51; NA: 2	*6.72E-01* ^3^
Clinical stage	iia: 0; iib: 0; iic: 0; iiia: 1; iiib: 0; iiic: 38; iv: 10; NA: 0	iia: 0; iib: 0; iic: 1; iiia: 0; iiib: 1; iiic: 24; iv: 3; NA: 1	iia: 0; iib: 0; iic: 3; iiia: 1; iiib: 3; iiic: 51; iv: 16; NA: 1	iia: 0; iib: 0; iic: 3; iiia: 4; iiib: 4; iiic: 22; iv: 7; NA: 1	iia: 0; iib: 1; iic: 1; iiia: 0; iiib: 2; iiic: 33; iv: 9; NA: 1	iia: 0; iib: 0; iic: 2; iiia: 1; iiib: 5; iiic: 38; iv: 6; NA: 0	iia: 1; iib: 1; iic: 2; iiia: 0; iiib: 4; iiic: 34; iv: 4; NA: 0	iia: 0; iib: 0; iic: 1; iiia: 0; iiib: 1; iiic: 42; iv: 12; NA: 0	iia: 2; iib: 2; iic: 4; iiia: 0; iiib: 1; iiic: 41; iv: 7; NA: 0	*2.65E-02* ^1^
Tumor residual disease	> 20 mm: 10; 1–10 mm: 26; 11–20 mm: 6; no macroscopic disease: 4; NA: 3	> 20 mm: 5; 1–10 mm: 17; 11–20 mm: 5; no macroscopic disease: 12; NA: 4	> 20 mm: 17; 1–10 mm: 29; 11–20 mm: 5; no macroscopic disease: 12; NA: 12	> 20 mm: 6; 1–10 mm: 18; 11–20 mm: 1; no macroscopic disease: 12; NA: 4	> 20 mm: 11; 1–10 mm: 21; 11–20 mm: 4; no macroscopic disease: 3; NA: 8	> 20 mm: 4; 1–10 mm: 24; 11–20 mm: 5; no macroscopic disease: 12; NA: 7	> 20 mm: 8; 1–10 mm: 15; 11–20 mm: 5; no macroscopic disease: 13; NA: 5	> 20 mm: 6; 1–10 mm: 29; 11–20 mm: 2; no macroscopic disease: 14; NA: 5	> 20 mm: 11; 1–10 mm: 25; 11–20 mm: 2; no macroscopic disease: 14; NA: 5	*6.13E-02* ^1^
Tumor tissue site	Omentum: 0; Ovary: 48; Peritoneum ovary: 1	Omentum: 0; Ovary: 30; Peritoneum ovary: 0	Omentum: 1; Ovary: 74; Peritoneum ovary: 0	Omentum: 0; Ovary: 41; Peritoneum ovary: 0	Omentum: 1; Ovary: 46; Peritoneum ovary: 0	Omentum: 0; Ovary: 52; Peritoneum ovary: 0	Omentum: 0; Ovary: 46; Peritoneum ovary: 0	Omentum: 0; Ovary: 56; Peritoneum ovary: 0	Omentum: 0; Ovary: 57; Peritoneum ovary: 0	*5.01E-01* ^3^
Venous invasion	No: 3; Yes: 3; NA: 43	No: 3; Yes: 10; NA: 17	No: 8; Yes: 7; NA: 60	No: 12; Yes: 3; NA: 26	No: 1; Yes: 10; NA: 36	No: 10; Yes: 5; NA: 37	No: 7; Yes: 20; NA: 19	No: 3; Yes: 1; NA: 52	No: 3; Yes: 10; NA: 44	*7.24E-02* ^3^
Vital status	Alive: 9; Dead: 40, NA: 0	Alive: 14; Dead: 16; NA: 0	Alive: 33; Dead: 42; NA:0	Alive: 18; Dead: 23; NA: 0	Alive: 20; Dead: 27; NA:	Alive: 20; Dead: 31; NA: 1	Alive: 28; Dead: 18; NA: 0	Alive: 31; Dead: 25; NA: 0	Alive: 27; Dead: 30; NA: 0	*1.90E-03* ^*3*^
Primary therapy outcome success	Complete remission/response: 24; Partial remission/response: 12; Progressive disease: 3; Stable disease: 1; NA: 9	Complete remission/response: 17; Partial remission/response: 3; Progressive disease: 4; Stable disease: 2; NA: 4	Complete remission/response: 41; Partial remission/response: 7; Progressive disease: 2; Stable disease: 4; NA: 21	Complete remission/response: 24; Partial remission/response: 4; Progressive disease: 2; Stable disease: 0; NA: 11	Complete remission/response: 24; Partial remission/response: 8; Progressive disease: 4; Stable disease: 3; NA: 8	Complete remission/response: 29; Partial remission/response: 6; Progressive disease: 1; Stable disease: 5; NA: 11	Complete remission/response: 27; Partial remission/response: 5; Progressive disease: 4; Stable disease: 6; NA: 4	Complete remission/response: 36; Partial remission/response: 4; Progressive disease: 7; Stable disease: 2; NA: 7	Complete remission/response: 35; Partial remission/response: 5; Progressive disease: 5; Stable disease: 1; NA: 11	*5.08E-01* ^1^
Days lived known	22,300 ± 4750	21,100 ± 3150	22,800 ± 3930	23,800 ± 4050	23,300 ± 3840	24,500 ± 4140	23,000 ± 4490	22,800 ± 4430	23,400 ± 4240	*3.85E-02* ^2^

Nominally statistically significant differences (*p* < 0.05) are shown in italic. Interestingly, significant differences are detected in lymphatic invasion, clinical stage at diagnosis, vital status and the overall number of days alive

### Biomarker identification

#### Enrichment analysis

In order to detect differentially expressed features that are specific to one group, each of the nine clusters was compared to the rest of the dataset. Table 3 shows the summary of statistically different features (*p*-value < 0.05, 5% FDR correction) identified in each comparison.

**Table 3:** Number of statistically significant different features obtained when comparing each cluster against all other patients in the dataset, for each platform. *P*-values were computed by a linear model in each ‘omics platform independently, and Benjamini-Hochberg FDR corrected

	1 vs Rest (49 vs 404)	2 vs Rest (30 vs 423)	3 vs Rest (75 vs 378)	4 vs Rest (41 vs 412	5 vs Rest (47 vs 406	6 vs Rest (52 vs 401	7 vs Rest (46 vs 407	8 vs Rest (56 vs 397	9 vs Rest (57 vs 396)
mRNA	1861	245	4101	1073	2480	3617	2557	4620	1843
Methylation	335	550	4	388	498	233	387	528	75
miRNA	18	0	1	9	24	1	8	14	11

Enrichment analysis of features differentially expressed/abundant between the clusters was then performed. Complete results are presented in the Additional file 2; an overview of results for which there is already evidence in the literature is presented below in Table 4.

**Table 4:** Enrichment analysis for each comparison across all ‘omics types, with q-values, and the literature references mentioning involvement of the terms in ovarian cancer development. Q-values are the minimal false discovery rate at which the test may be called significant, or in other words, the *p*-value threshold to satisfy the FDR criteria set by the Benjamini-Hochberg procedure

Term	Term type	‘Omic type	Contrast	q-value	Reference of implication in ovarian cancer
E2F	Transcription factor	Transcriptomics	1 vs Rest	8.17E-48	[123, 124]
Sp1	Transcription factor	Transcriptomics	1 vs Rest	1.95E-35	[125]
Mitochondrial translation	Reactome	Transcriptomics	1 vs Rest	9.02E-21	[126]
hsa-miR-193a-5p	miRNA	Transcriptomics	1 vs Rest	4.33E-09	[127]
CREM	Transcription factor	Methylation	1 vs Rest	2.45E-03	[128]
hsa-miR-940	miRNA	Transcriptomics	1 vs Rest	6.80E-03	[129]
hsa-miR-601	miRNA	Transcriptomics	1 vs Rest	6.81E-03	[129]
hsa-miR-503	miRNA	Transcriptomics	1 vs Rest	1.41E-02	[129]
AP-1	Transcription factor	Methylation	1 vs Rest	1.52E-02	[130]
TCF-4	Transcription factor	Methylation	1 vs Rest	2.04E-02	[131]
hsa-miR-361-3p	miRNA	Transcriptomics	1 vs Rest	2.53E-02	[129]
C/EBP	Transcription factor	Methylation	2 vs Rest	1.13E-05	[132]
LMXB1	Transcription factor	Methylation	2 vs Rest	9.32E-05	[133]
hsa-miR-330-5p	miRNA	Transcriptomics	2 vs Rest	7.57E-03	[134]
Chemical carcinogenesis	KEGG pathways	Transcriptomics	2 vs Rest	1.77E-02	[135–137]
hsa-miR-335	miRNA	Transcriptomics	2 vs Rest	3.95E-02	[138]
MZF-1	Transcription factor	Transcriptomics	3 vs Rest	4.06E-39	[139]
SREBP-1	Transcription factor	Transcriptomics	3 vs Rest	5.29E-38	[140]
AP-2gamma	Transcription factor	Transcriptomics	3 vs Rest	1.79E-36	[141]
GPCR ligand binding	Reactome	Transcriptomics	3 vs Rest	8.14E-10	[142]
hsa-miR-328	miRNA	Transcriptomics	3 vs Rest	9.92E-10	[129]
hsa-miR-370	miRNA	Transcriptomics	3 vs Rest	1.09E-08	[129]
hsa-miR-601	miRNA	Transcriptomics	3 vs Rest	1.07E-07	[129]
hsa-miR-423-5p	miRNA	Transcriptomics	3 vs Rest	1.36E-06	[129]
hsa-miR-139-3p	miRNA	Transcriptomics	3 vs Rest	2.28E-05	[129]
hsa-miR-769-5p	miRNA	Transcriptomics	3 vs Rest	9.05E-05	[129]
hsa-miR-339-3p	miRNA	Transcriptomics	3 vs Rest	2.16E-04	[129]
hsa-miR-940	miRNA	Transcriptomics	3 vs Rest	2.94E-04	[129]
hsa-miR-542-5p	miRNA	Transcriptomics	3 vs Rest	8.13E-04	[129]
hsa-miR-483-5p	miRNA	Transcriptomics	3 vs Rest	1.50E-03	[129]
hsa-miR-361-3p	miRNA	Transcriptomics	3 vs Rest	7.88E-03	[129]
hsa-miR-449a	miRNA	Transcriptomics	3 vs Rest	4.87E-02	[129]
T cell aggregation	GO Biological Process	Transcriptomics	4 vs Rest	1.94E-38	[143]
T cell activation	GO Biological Process	Transcriptomics	4 vs Rest	1.94E-38	[144]
Natural killer cell mediated cytotoxicity	KEGG pathways	Transcriptomics	4 vs Rest	8.60E-14	[145]
Cell adhesion molecules (CAMs)	KEGG pathways	Transcriptomics	4 vs Rest	2.37E-11	[146]
Hedgehog ‘on’ state	Reactome	Transcriptomics	4 vs Rest	7.21E-05	[147]
HIC1	Transcription factor	Methylation	4 vs Rest	2.46E-04	[148]
hsa-miR-328	miRNA	Transcriptomics	4 vs Rest	1.49E-02	[129]
AP-2gamma	Transcription factor	Transcriptomics	4 vs Rest	3.00E-02	[141]
T cell activation	GO Biological Process	Transcriptomics	5 vs Rest	1.94E-38	[144]
T cell aggregation	GO Biological Process	Transcriptomics	5 vs Rest	2.25E-22	[143]
Natural killer cell mediated cytotoxicity	KEGG pathways	Transcriptomics	5 vs Rest	8.60E-14	[145]
Antigen processing and presentation	KEGG pathways	Transcriptomics	5 vs Rest	4.33E-11	[149]
Interferon alpha/beta signalling	Reactome	Transcriptomics	5 vs Rest	6.11E-08	[150]
hsa-miR-423-5p	miRNA	Transcriptomics	5 vs Rest	3.09E-05	[129]
hsa-miR-328	miRNA	Transcriptomics	5 vs Rest	5.23E-04	[129]
VEGFA-VEGFR2 Pathway	Reactome	Transcriptomics	5 vs Rest	2.57E-03	[151, 152]
Hedgehog ‘off’ state	Reactome	Transcriptomics	5 vs Rest	1.21E-02	[153]
hsa-miR-139-3p	miRNA	Transcriptomics	5 vs Rest	1.35E-02	[129]
NF- κB signalling pathway	KEGG pathways	Transcriptomics	5 vs Rest	1.53E-02	[154]
hsa-miR-601	miRNA	Transcriptomics	5 vs Rest	2.71E-02	[129]
Jak-STAT signalling pathway	KEGG pathways	Transcriptomics	5 vs Rest	3.54E-02	[155]
hsa-miR-375	miRNA	Transcriptomics	5 vs Rest	3.74E-02	[129]
Signalling by GPCR	Reactome	Transcriptomics	6 vs Rest	1.24E-14	[156]
hsa-miR-328	miRNA	Transcriptomics	6 vs Rest	1.47E-08	[129]
hsa-miR-601	miRNA	Transcriptomics	6 vs Rest	6.94E-07	[129]
hsa-miR-370	miRNA	Transcriptomics	6 vs Rest	2.46E-06	[129]
hsa-miR-423-5p	miRNA	Transcriptomics	6 vs Rest	4.81E-06	[129]
hsa-miR-423-3p	miRNA	Transcriptomics	6 vs Rest	1.77E-05	[129]
cAMP metabolic process	GO Biological Process	Transcriptomics	6 vs Rest	9.22E-05	[157]
hsa-miR-769-5p	miRNA	Transcriptomics	6 vs Rest	5.13E-04	[129]
hsa-miR-139-3p	miRNA	Transcriptomics	6 vs Rest	2.70E-03	[129]
hsa-miR-483-5p	miRNA	Transcriptomics	6 vs Rest	4.90E-03	[129]
hsa-miR-940	miRNA	Transcriptomics	6 vs Rest	5.05E-03	[129]
T cell selection	GO Biological Process	Transcriptomics	6 vs Rest	1.41E-02	[158]
Arachidonic acid metabolism	KEGG pathways	Transcriptomics	6 vs Rest	1.42E-02	[135]
hsa-miR-542-5p	miRNA	Transcriptomics	6 vs Rest	1.73E-02	[129]
Oxidative phosphorylation	KEGG pathways	Transcriptomics	7 vs Rest	9.49E-13	[159]
Stabilization of p53	Reactome	Transcriptomics	7 vs Rest	1.06E-07	[160]
Spliceosome	KEGG pathways	Transcriptomics	7 vs Rest	1.59E-07	[161]
NF-kB signalling pathway	Reactome	Transcriptomics	7 vs Rest	3.97E-05	[154]
hsa-miR-542-5p	miRNA	Transcriptomics	7 vs Rest	2.53E-03	[129]
hsa-miR-601	miRNA	Transcriptomics	7 vs Rest	2.62E-03	[129]
hsa-miR-423-5p	miRNA	Transcriptomics	7 vs Rest	5.88E-03	[129]
hsa-let-7c	miRNA	Transcriptomics	7 vs Rest	2.67E-02	[129]
Regulation of HIF by oxygen	Reactome	Transcriptomics	7 vs Rest	3.32E-02	[162]
hsa-miR-361-3p	miRNA	Transcriptomics	7 vs Rest	4.16E-02	[129]
hsa-miR-328	miRNA	Transcriptomics	8 vs Rest	9.25E-15	[129]
hsa-miR-370	miRNA	Transcriptomics	8 vs Rest	3.60E-11	[129]
hsa-miR-940	miRNA	Transcriptomics	8 vs Rest	1.37E-10	[129]
hsa-miR-423-5p	miRNA	Transcriptomics	8 vs Rest	4.29E-10	[129]
hsa-miR-423-3p	miRNA	Transcriptomics	8 vs Rest	7.47E-09	[129]
hsa-miR-139-3p	miRNA	Transcriptomics	8 vs Rest	5.08E-07	[129]
hsa-miR-601	miRNA	Transcriptomics	8 vs Rest	9.47E-07	[129]
hsa-miR-542-5p	miRNA	Transcriptomics	8 vs Rest	4.72E-04	[129]
hsa-miR-361-3p	miRNA	Transcriptomics	8 vs Rest	1.07E-03	[129]
hsa-miR-483-5p	miRNA	Transcriptomics	8 vs Rest	1.32E-03	[129]
hsa-miR-769-5p	miRNA	Transcriptomics	8 vs Rest	1.68E-03	[129]
Potassium signalling pathway	Reactome	Transcriptomics	8 vs Rest	1.15E-02	[163]
hsa-miR-99b	miRNA	Transcriptomics	8 vs Rest	1.93E-02	[129]
hsa-miR-339-3p	miRNA	Transcriptomics	8 vs Rest	2.28E-02	[129]
T cell lineage commitment	GO Biological Process	Transcriptomics	8 vs Rest	3.80E-02	[164]
hsa-miR-139-3p	miRNA	Transcriptomics	9 vs Rest	3.58E-09	[129]
hsa-miR-423-5p	miRNA	Transcriptomics	9 vs Rest	5.89E-09	[129]
hsa-miR-328	miRNA	Transcriptomics	9 vs Rest	2.32E-08	[129]
hsa-miR-370	miRNA	Transcriptomics	9 vs Rest	4.83E-08	[129]
hsa-miR-423-3p	miRNA	Transcriptomics	9 vs Rest	3.89E-06	[129]
hsa-miR-940	miRNA	Transcriptomics	9 vs Rest	5.37E-06	[129]
hsa-miR-769-5p	miRNA	Transcriptomics	9 vs Rest	1.07E-04	[129]
hsa-miR-339-3p	miRNA	Transcriptomics	9 vs Rest	0.000173	[129]
hsa-miR-601	miRNA	Transcriptomics	9 vs Rest	2.05E-04	[129]
hsa-miR-483-5p	miRNA	Transcriptomics	9 vs Rest	7.33E-03	[129]
Calcium signalling pathway	KEGG pathways	Transcriptomics	9 vs Rest	1.55E-02	[165]
hsa-miR-542-5p	miRNA	Transcriptomics	9 vs Rest	1.69E-02	[129]
cAMP signalling pathway	KEGG pathways	Transcriptomics	9 vs Rest	2.33E-02	[166]
Ion transfer	GO Biological Process	Transcriptomics	9 vs Rest	3.43E-02	[167]

In short, the biological functions enriched in each cluster are as follows: cluster 1 is mostly enriched in mitochondrial translation and energy metabolism, cell cycle regulation, negative regulation of apoptosis and DNA damage response. In addition, several miRNAs and transcription factors are enriched; the details can be found in the Additional file 2.

Cluster 2 is associated with chemical carcinogenesis, miR-330-5p, miR-693-5p and the Pax-2 transcription factor. Other transcription factors are also highlighted through the methylation measurements.

Cluster 3 is associated with immune system regulation (T cell-related processes, and more precisely CD4 and CD8-T cells lineages-related processes…), cell-cell signalling, cAMP signalling, cytokine-cytokine interaction, G-Protein coupled receptor (GPCR) ligand binding and neuronal and muscle-related pathways (potassium and calcium channels, other ion channels and synapses). Again, several miRNAs and transcription factors are highlighted.

Cluster 4 is also associated with the immune response, and key functions such as lymphocyte activation, T cell aggregation, differentiation, proliferation and activation, adaptive immune system, regulation of lymphocyte cell-cell activation, immune response-regulating signalling pathway, cytokine-cytokine receptor interaction, antigen processing and presentation, hematopoietic cell lineage and hematopoiesis and B cell activation. Primary immunodeficiency pathway and cell adhesion molecules, along with miR-938 and several transcription factors are also enriched.

Cluster 5 is related to immune response, enriched in lymphocyte activation, T cell aggregation, differentiation, activation and proliferation, leukocyte differentiation, aggregation and activation, positive regulation of cell-cell adhesion, antigen processing and presentation, cytokine production, inflammatory response, NK cell-mediated cytotoxicity and cytokine-cytokine receptor interaction. Other processes involved are NF-κB signalling, Jak-STAT signalling, Interferon α/β signalling, TCR signalling, VEGF signalling, VEGFR2-mediated cell proliferation, Hedgehog ‘off’ state, along with several miRNAs and transcription factors.

Cluster 6 is enriched in several signalling pathways, such as cAMP, GPCR signalling, arachidonic acid metabolism and fatty acids metabolism, as well as positive T cell selection, several miRNAs and transcription factors.

Cluster 7 is linked with respiratory metabolism, p53 and cell cycle regulation, splicing regulation as well as signalling by NF-κB and miRNAs and transcription factors.

Cluster 8 is enriched with T cell lineage commitment, potassium channels, miRNAs and transcription factors.

Cluster 9 is associated with ion transport (including synaptic, calcium and potassium channels), cAMP signalling, nicotine addiction, as well as miRNAs and transcription factors.

Each cluster is linked with one or several of the well-known hallmarks of cancer such as regulation of the cell cycle (clusters 1 and 7), energy metabolism (cluster 1 and 7), immune system (clusters 3, 4, 5 and 8), epithelial-to-mesenchymal transition (cluster 4) or angiogenesis (cluster 5) [104–106]. Interestingly, our analysis based on ‘omics profiles is able to identify clusters that seem to separate some of those hallmarks out, while an analysis taking into account only the clinical data cannot. As seen above, cluster 6 is associated with a higher rate of survival. It would therefore be interesting to further explore the signalling networks enriched in the comparison between cluster 6 and the other clusters to identify the molecular mechanisms responsible for the extended survival.

#### Machine-learning predictive modelling

The next step in the analysis is to establish a model that can predict which cluster a patient belongs to, based on the ‘omics measurements alone. Machine-learning techniques (reviewed in [107, 108]), available in the caret R package [109] and in the MixOmics R packages [110, 111] were used.

Two models were built in parallel, on the same dataset.

1. A Recursive Feature Elimination (RFE) procedure was performed to identify the smallest number of features from the three ‘omics platforms that allow satisfactory separation of the clusters. This procedure was controlled by Leave-Group-Out Cross Validation (LGOCV) with 100 iterations (this number was chosen to ensure convergence of the validation procedure) and using between 1 and 50 predictors, with the addition of the whole set of 6753 features. A Random Forest (RF) model was built with the features identified in the previous step. To avoid overfitting, the RF model was built using LGOCV with 100 iterations and in three quarters of the samples available (*N* = 300) and then tested in the remaining quarter of samples (*N* = 153). More details can be found in the Additional file 3.
2. Concatenation-based integration of data combines multiple datasets into a single large dataset, with the aim to predict an outcome. However, this approach does not account for or model relationships between datasets and thus limits our understanding of molecular interactions at multiple functional levels. This is the rationale behind the development of novel integrative modelling methods, such as the DIABLO sPLSDA method [112]. A DIABLO model was built using the same dataset as the SNF analysis described above. A DIABLO model is a type of partial least square (sparse PLS Discriminant Analysis) regression model, which uses multiple ‘omics platform measurements on the same samples to predict an outcome, with a biomarkers selection step (sparse) to select necessary and sufficient features to predict the groups (discriminant analysis) within the outcome. Details of this analysis can be found in the Additional file 4. In short, this analysis was run as follows: the datasets were split in 2/3 training and 1/3 testing sets. The DIABLO model was then trained with boundaries set on the number of features allowed per component (gene expression and methylation between 50 and 110 features, and between 5 and 35 miRNA features). The performances were then estimated within the training model by 10 repeats of 10-fold validation and the prediction power estimated in the testing set.

### Topological data analysis

**Fig. 7:**
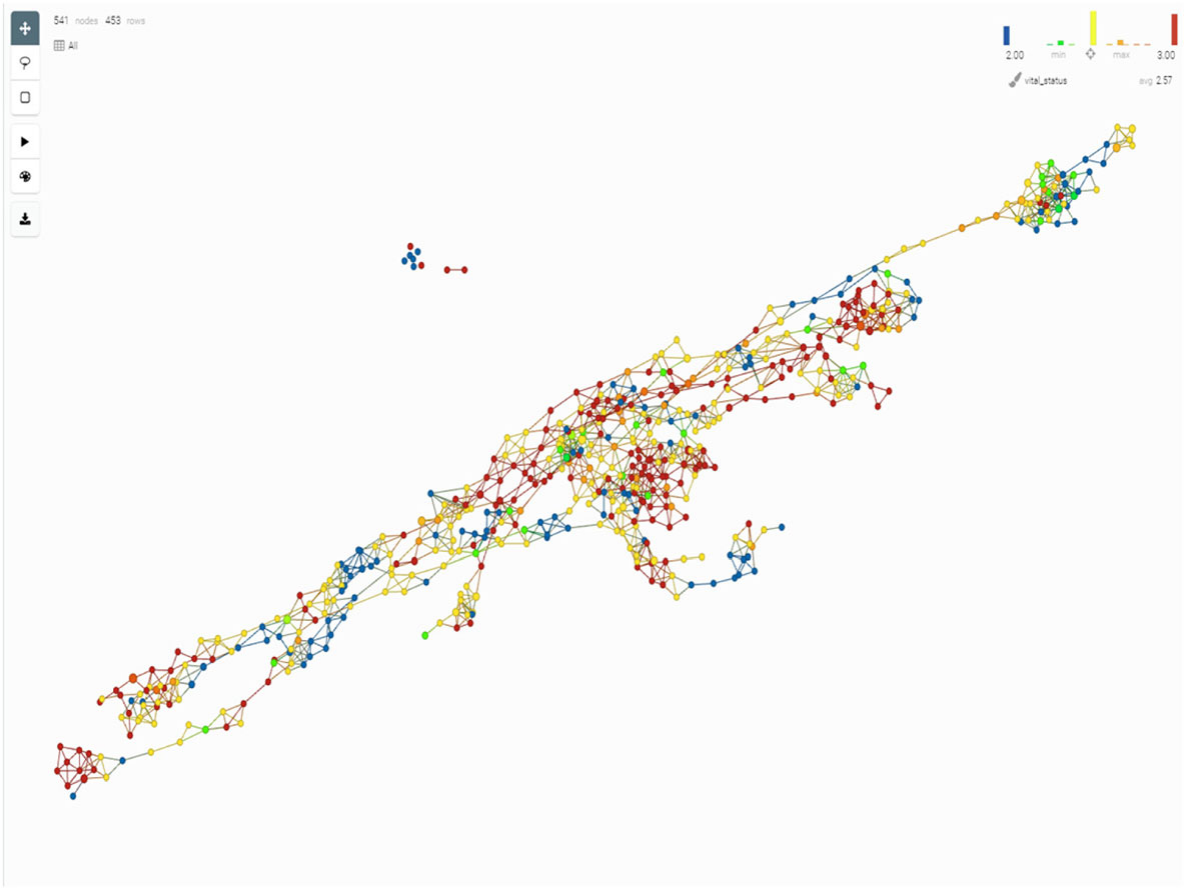
Network of patients shown in the TDA platform. The network is constructed as ‘bins’ grouping patients who are similar based on their ‘omics profiles. Each dot in the network represents a bin. The bins are overlapping by an adaptable percentage, and if at least one patient is present in the overlap of two bins, the two bins will be linked in the network. The survival status of the patients is then translated as a color scheme (blue representing deceased patients and red alive patients). Using this technique, it is easy to identify ‘islands’ of good and poor survival among the patients, and equally easy to acknowledge that there are more such islands than is identified through the clustering technique. Thorough analysis of such networks can lead to insights into biology, as detailed in [168]

In order to visualize the patients’ relationships as measured by their ‘omics profiles, we used Topology Data Analysis (TDA), a general framework to analyse high-dimensional, incomplete and noisy data in a manner that is less sensitive to the particular metric that is chosen, and provides dimensionality reduction and robustness to noise. TDA is embedded in the software produced by the Ayasdi company to which the data were uploaded [113]. As shown in Fig. 7, the network of patients’ similarities obtained through TDA analysis and then colored by the vital status of the patients at the end of the study shows a higher level of complexity than is identified by the clustering analysis, suggesting that statistical and/or technical limitations of the clustering methods prevent us to accurately represent reality.

## Discussion

Multi-omics data integration is, among other components of biological data integration, a very promising and emerging field. We show a structured and effective way to combine ‘omics data from multiple sources to search for molecular profiles of patients. This process allowed for the classification of a well-studied dataset of OV. Other studies have been performed, either on this same dataset [114–118], or on the same disease [119].

Tothill et al. in 2015 identified six clusters of patients, based on mRNA, immunohistochemistry and clinical data from a cohort of 285 Australian and Dutch participants, with a consensus clustering analysis of mRNA data alone. The TCGA consortium produced their own dataset in 2011, identifying four clusters based on combined mRNA, miRNA and DNA methylation data (data combined by summarising to the gene-level all datasets through a factor analysis) and using a non-negative matrix factorisation to identify clusters [120]. Further analysis of the same dataset was then performed by Zhang et al. [118], Jin et al. [115] and Kim et al. [116] (with some variations), but these authors did not look for new phenotypes in their analysis, rather comparing data based on clinical endpoints (survival time, histological grades and stage of disease). Gevaert et al. [114] used an original algorithm to combine DNA methylation, Copy Number Variation (CNV) and gene expression data, using the clusters defined in the TCGA original paper. Those studies showed different ways of analysing the data, leading to the identification of clinically relevant clusters in the case of Tothill and TCGA original paper [117, 119]. It is however the first time in this paper that TCGA mRNA, miRNA and methylation data were fused with an advanced data integration method to identify robust subtypes of disease.

The number of clusters found in the same dataset differs between the TCGA analysis and our analysis. We believe that the higher number of clusters we found is the result of more up-to-date and powerful methods for subtype discovery, as shown in the SNF original paper [55]. Moreover, the subtypes identified in this analysis do allow for a more in-depth classification of patients linked with specific molecular subtypes than was previously reported. Building predictive models based on multiple ‘omics profiles also contributes to the novelty of this approach as other reported studies did not produce such a model, with the exception of the Tothill et al. study [119] in which the authors developed a class prediction model based on transcriptomics data only.

Clinically speaking, classifications are most useful when they allow the identification of a subset of patients with a clinically relevant outcome, such as low or high survival rate, thus indicating where efforts may be focused to develop new drugs, therapies and procedures. In our analysis, the groups identified after feature reduction are statistically different in terms of survival rate and time. For example, cluster 6 shows the highest rate of survival among the 9 clusters identified and is associated with the GPCR signalling pathway, cAMP, ion channels, arachidonic acid metabolism and a number of miRNAs (see Table 4 or the Additional file 2 for more details).

Interestingly, while the two sets of groups defined with or without feature reduction show differences in invasion and clinical stage, statistically significant differences in vital status are only detected amongst groups defined with feature reduction. The reduced data also allows for the definition of a higher number of stable groups (9 instead of 4), thereby pointing to the usefulness of performing feature reduction prior to clustering analysis.

The biological functions highlighted by enrichment analysis between the clusters indicate that these are associated with different biological mechanisms leading to the development of cancer in patients, ranging from immune system disorders, cell cycle dysregulation, impaired response to DNA damage, modified energy metabolism, etc.

The predictive models that were trained and tested with two different methods gave mixed power results. In the Random Forest case, the model could predict quite well when patients did not belong to the clusters, but not so well when patients did belong to them; in other words, the model is specific but not sensitive. In the case of the DIABLO PLS, the model is able to predict fairly accurately the clusters 4 and 8 and less accurately cluster 5. Moreover, in the case of the DIABLO analysis, the model showed that the clusters have different ‘omics patterns, with clusters 2 and 8 showing distinct methylation profiles, and cluster 4 showing different methylation and transcriptomics profiles.

The results presented in this manuscript are not perfectly predictive, however. It seems that the cluster definitions are not as stable as they could be; the predictive models are not accurate in all clusters and the survival status of the clusters are not clear cut. This reflects the fact shown in Fig. 7, that there seems to be much more complexity within the dataset than what the clustering analysis is able to detect.

This is due to multiple factors: the recurring issue of low number of patients, which in turn influences the number of clusters we can find with statistical confidence – a point which is not taken into account in the TDA analysis discussed here – and highlighting the need for better stratification methods in the context of personalized medicine where, ideally, each patient is his/her own cluster (*n* = 1); sub-optimal clustering methods and algorithms also play a part in this result and it is our hope that continuous methods development will allow for better classification. Clustering analysis is descriptive in nature: applying a clustering algorithm to a dataset will always yield clusters, whether real clusters exist or not. Analytical methods exist to ascertain cluster ‘reality’, among which stability in patients through bootstrapping, stability in time through cluster identification from time-series experiments [121], meta clustering across several studies, yet only replication studies may confirm the existence of these clusters. Such replication effort however lies outside the scope of this manuscript.

Despite the use of most recent databases and tools, the biological interpretation of the differences between the clusters remains challenging. The main issues stem from the overlapping nature of pathways described in literature and the non-unicity of relationships between biological entities, leading to a high false positive rate in the results of pathway analysis [97]. Efforts are made in the systems biology community to correct these shortcomings, among which the disease maps mentioned above.

This underlines the variability in biological events potentially leading to the development of cancer and metastasis and the need for a more personalised care for patients suffering from complex diseases, such as cancer. It is our hope that this methodology will be repeated on other datasets, diseases and clinical situations as it is one more step towards establishing a true personalised data analysis pipeline.

The clusters that were found in this analysis are interesting hypotheses. They would however require further validation to become clinically useful, as detailed in the replication of findings section above. We encourage other researchers to use our findings in their research towards a cross-validated and clinically useful stratification of ovarian cancer, towards a better and more personalized care.

## Conclusion

This article presents an overview of the integrative systems biology analyses developed, performed and validated in the IMI U-BIOPRED and eTRIKS projects, proposing a template for other researchers wishing to perform similar analyses for other diseases. We demonstrate the usefulness of generating hypotheses through a fingerprint/handprint analysis by applying to a well-studied dataset of ovarian carcinoma, identifying a higher number of robust groups than previously reported, potentially improving our understanding of this disease. Better characterisation of the clusters found in the handprint analyses and validation of the predictive model obtained by machine learning are both ongoing. We believe that handprint analyses, performed on large scale ‘omics datasets will allow researchers to identify subtypes of disease (phenotypes and endotypes) [34] with greater confidence, providing better diagnosis tools for the clinicians, new avenues for drug development for the pharmaceutical industry and deeper insights into disease mechanisms. To be effective, handprint analyses need to be performed on the same subjects with multiple ‘omics platforms. They suffer from some limitations, such as the decreasing but nevertheless still elevated cost of ‘omics data production and the protocol standardisation requirements to avoid time-consuming data pre-processing, the rather large technical, human resources and expertise requirements to perform the analyses (particularly the machine-learning analysis) or the lack of accurate and independent benchmarking tools to identify the most powerful and/or best-suited method to analyse a particular dataset.

Additional work is therefore needed to make the framework and the analyses proposed here more accessible to a broad audience of health researchers. Efforts of the bioinformatics community are shifting in this direction; for instance, the eTRIKS European project (http://www.etriks.org) or the Galaxy project hosted in the USA (https://galaxyproject.org) mandate the delivery of user-friendly interfaces to advanced bioinformatics resources. Implementation of P4 medicine across the entire health spectrum [122] will be leveraged through promotion of advanced analytical tools available to the larger multidisciplinary community. The methods and results demonstrated in this paper should contribute to pave this promising road.

## Additional files

Additional file 1: AUC of consensus clustering. (XLSX 13 kb)

Additional file 2: Complete results of the enrichment analysis between clusters. (XLSX 4293 kb)

Additional file 3: **Table S7.** Estimated accuracy and standard deviation of the RFE procedure. **Table S8.** Accuracy and Kappa values of the Random Forest models in the training set. **Table S9.** Performances values for the Random Forest model in the testing set. **Figure S11.** Relative importance of the top 20 predictors building the final model of the RF. The importance axis is scaled, with the mRNA expression of CD3D scaled to 100% and the methylation state of POLA2 to 0% (not shown). (DOCX 18 kb)

Additional file 4: DIABLO sPLSDA model results. (DOCX 18966 kb)
