# Supplementary material for: A computational framework for complex disease stratification from multiple large-scale datasets
Source: BMC Syst Biol. 2018 May 29;12:60. doi: 10.1186/s12918-018-0556-z (PMC5975674; doi:10.1186/s12918-018-0556-z)
Supplement: Supplementary file 3 — Table S7. Estimated accuracy and standard deviation of the RFE procedure. Table S8. Accuracy and Kappa values of the Random Forest models in the training set. Table S9. Performances values for the Random Forest model in the testing set. Figure S11. Relative importance of the top 20 predictors building the final model of the RF. The importance axis is scaled, with the mRNA expression of CD3D scaled to 100% and the methylation state of POLA2 to 0% (not shown). (DOCX 18 kb) [file 12918_2018_556_MOESM3_ESM.docx]

# RFE model results.

## Choice of number of relevant features and model training:

Results were as follows: the RFE showed, as expected, that using the full dataset gave the best prediction however, a smaller number of features did not tremendously decrease the estimated accuracy. In order to limit over-fitting, a maximum of 45 predictors were chosen (see Table 7 for summary; full table available in the additional file 6, with the list of 45 predictors selected).

Table 7: Estimated accuracy and standard deviation of the RFE procedure.

| Number of variables | Global accuracy | Accuracy SD |
| --- | --- | --- |
| 45 | 0.4694 | 0.04871 |
| 6753 | 0.7500 | 0.04215 |

Table 7: Estimated accuracy with standard deviation across the resampling iterations of the RFE procedure. At each iteration a smaller number of features is selected and tested for predictive power against the real cluster associations provided to the algorithm.

The 45 predictors were then used to build a RF model in the training set (300 samples). Again, RF tries to build models by varying the number of predictors (between 2 and the number identified at the previous step) and the highest accuracy model was chosen (see Table 8).

Table 8: Accuracy and Kappa values of the Random Forest models in the training set

| Number of features | Accuracy | Kappa |
| --- | --- | --- |
| 2 | 0.54 | 0.48 |
| 23 | 0.57 | 0.52 |
| 45 | 0.57 | 0.51 |

Table 8: Accuracy and Kappa values of the RF models on the *training* set by the number of predictors considered. RF automatically chooses 45 predictors as the optimal number. As this algorithm is different from the one used to estimate the number of useful predictors, the accuracy values differ slightly. The Kappa value (Cohen’ Kappa) is a measure of agreement between two observations of categorical variables.

Testing of the model

When assessing the model with 45 predictors in the *testing* set, the overall accuracy was of 0.529. Details of prediction performances are found in Table 9. Specificity of the model is overall very large (above 0.88), whereas sensitivity is variable (between 0.26 and 0.89).

Table 9: Performances values for the Random Forest model in the testing set

|  | Cluster 1 | Cluster 2 | Cluster 3 | Cluster 4 | Cluster 5 | Cluster 6 | Cluster7 | Cluster 8 | Cluster 9 |
| --- | --- | --- | --- | --- | --- | --- | --- | --- | --- |
| Sensitivity | 0.58 | 0.67 | 0.33 | 0.62 | 0.53 | 0.50 | 0.26 | 0.89 | 0.45 |
| Specificity | 0.88 | 0.99 | 0.91 | 0.98 | 0.90 | 0.91 | 0.96 | 0.96 | 0.98 |
| Positive predictive value | 0.29 | 0.91 | 0.37 | 0.81 | 0.41 | 0.35 | 0.54 | 0.77 | 0.62 |
| Negative predictive value | 0.96 | 0.96 | 0.89 | 0.94 | 0.94 | 0.95 | 0.88 | 0.98 | 0.96 |
| Prevalence | 0.078 | 0.098 | 0.14 | 0.14 | 0.11 | 0.091 | 0.15 | 0.12 | 0.072 |
| Detection rate | 0.046 | 0.065 | 0.046 | 0.085 | 0.059 | 0.046 | 0.039 | 0.11 | 0.033 |
| Detection prevalence | 0.16 | 0.072 | 0.12 | 0.10 | 0.14 | 0.13 | 0.072 | 0.14 | 0.052 |
| Balanced accuracy | 0.73 | 0.83 | 0.62 | 0.80 | 0.72 | 0.70 | 0.61 | 0.93 | 0.72 |

Table 9: Performance values for the RF model in each of the 9 clusters of patients, using 45 predictors and evaluated in the testing set of patients (153 patients).

The relative importance of the predictors when building the model is described in Figure 11. The five most important predictors of this model are the mRNA expression of CD3D, c1orf86, PRR7, F12 as well as the methylation state of ANXA5.

Figure 11: Relative importance of the top 20 predictors building the final model of the RF. The importance axis is scaled, with the mRNA expression of CD3D scaled to 100% and the methylation state of POLA2 to 0% (not shown).

CD3D is a T-cell surface marker, involved in T-cell development and signal transduction. CD3D expression in cancer cells is a sign of lymphocyte infiltration and is linked with higher patient survival in several cancer types (melanoma, ovarian, head and neck, breast, urothelial, colorectal, lung, hepatocellular, gall-bladder and oesophageal) {Angell, 2013 #1571}. CD3D has been associated with a cluster of good prognostic patients in a larger cohort of breast cancer patients {Curtis, 2012 #1581}, and similar observations have been made in ovarian cancer {Leffers, 2010 #1592}.

C1orf86 (or FAAP20, Fanconi Anaemia Core Complex Associated Protein 20) is part of a 14 proteins complex recruited to the sites of DNA interstrand crosslink damages. Defects in this repair mechanism can cause the development and progression of cancers {Deans, 2011 #1596}. Inactivation of the Fanconi anaemia pathway causes the Fanconi anaemia disease, which is a predisposition for cancer {Ceccaldi, 2016 #1598}.

PRR7 (Proline-Rich 7 (synaptic) is a potential regulator of signalling and apoptosis in activated T cells {Hrdinka, 2011 #1604}.

F12 (or Coagulation factor XII) is found upregulated in epithelial ovarian cancer {Wang, 2005 #1605}. F12 can trigger a macrophage invasion in tumour environments which promotes metastasis {Musrap, 2012 #1606}, and is linked with monocyte/macrophage mediating peritoneal metastasis of epithelial ovarian cancer {Wang, 2010 #1607}.

The importance of ANXA5 methylation status is unclear. While Annexin V is routinely used as an assay to detect apoptotic cells, no information on the functional implication of its methylation status in (ovarian) cancer has been described.

Taken together, these predictors indicate that immune cells infiltration and the state of the DNA repair mechanism are the most important parameters in separating our clusters, and might be linked with survival of ovarian cancer patients, as also indicated in recent literature in the general context of cancer {Gajewski, 2013 #1610;Jochems, 2011 #1608}, and in ovarian carcinoma in particular, where presence of T-cell infiltration in tumours in shown to correlate with survival time (patients whose tumours contained T cells had a median duration of progression-free survival double than those whose tumours did not contain T cells {Sato, 2005 #1611;Zhang, 2003 #1613}).
