# Supplementary material for: A computational framework for complex disease stratification from multiple large-scale datasets
Source: BMC Syst Biol. 2018 May 29;12:60. doi: 10.1186/s12918-018-0556-z (PMC5975674; doi:10.1186/s12918-018-0556-z)
Supplement: Supplementary file 4 — DIABLO sPLSDA model results. (DOCX 18966 kb) [file 12918_2018_556_MOESM4_ESM.docx]

# DIABLO sPLSDA model results

## Training the model

The first step of feature selection was performed, similar to what was used in the RF models, with the difference is that the feature reduction steps in this method is performed for each component of the PLS model, instead of a single list of informative features. The number of component was set as 8, being the number of groups in the outcome vector -1.

The boundaries on the number of features allowed per component were set as:

- Gene expression and methylation between 50 and 110 features;
- miRNA between 5 and 35 features.

The model was then trained with a 5-fold cross validation, with all combinations of selected features allowed.

The number of features selected per feature with the highest performances is shown in the table below.

|  | Comp 1 | Comp 2 | Comp 3 | Comp 4 | Comp 5 | Comp 6 | Comp 7 | Comp 8 |
| --- | --- | --- | --- | --- | --- | --- | --- | --- |
| Gene Expression | 65 | 50 | 110 | 95 | 80 | 70 | 110 | 60 |
| Methylation | 70 | 95 | 100 | 90 | 50 | 60 | 75 | 100 |
| miRNA | 25 | 15 | 30 | 20 | 15 | 20 | 20 | 30 |

Performances of the final model were then evaluated by 10 repeats on 10-fold cross validation and estimated as follows.

|  | Comp 1 | Comp 2 | Comp 3 | Comp 4 | Comp 5 | Comp 6 | Comp 7 | Comp 8 |
| --- | --- | --- | --- | --- | --- | --- | --- | --- |
| CL1 | 1 | 1 | 0.465 | 0.462 | 0.473 | 0.444 | 0.421 | 0.429 |
| CL2 | 1 | 1 | 1 | 0.988 | 0.896 | 0.884 | 0.868 | 0.848 |
| CL3 | 1 | 1 | 0.992 | 1 | 0.994 | 0.986 | 0.983 | 0.981 |
| CL4 | 0.961 | 0.0278 | 0.0694 | 0.0806 | 0.0666 | 0.05 | 0.058 | 0.0583 |
| CL5 | 0.897 | 0.131 | 0.2 | 0.223 | 0.243 | 0.210 | 0.182 | 0.177 |
| CL6 | 1 | 1 | 1 | 0.997 | 0.983 | 0.989 | 0.969 | 0.949 |
| CL7 | 0.561 | 0.982 | 0.969 | 0.651 | 0.610 | 0.441 | 0.385 | 0.403 |
| CL8 | 0.0787 | 0.0484 | 0.0879 | 0.0788 | 0.0879 | 0.109 | 0.109 | 0.139 |
| CL9 | 1 | 1 | 1 | 1 | 1 | 0.978 | 0.926 | 0.655 |
| Overall.ER | 0.825 | 0.667 | 0.623 | 0.585 | 0.573 | 0.541 | 0.520 | 0.498 |
| Overall.BER | 0.833 | 0.687 | 0.642 | 0.609 | 0.595 | 0.565 | 0.544 | 0.515 |

A graphical representation of the information contained in each component can be seen in scatter plots combined with correlation between datasets (‘blocks’), and with the loadings of features.

Component 1

| 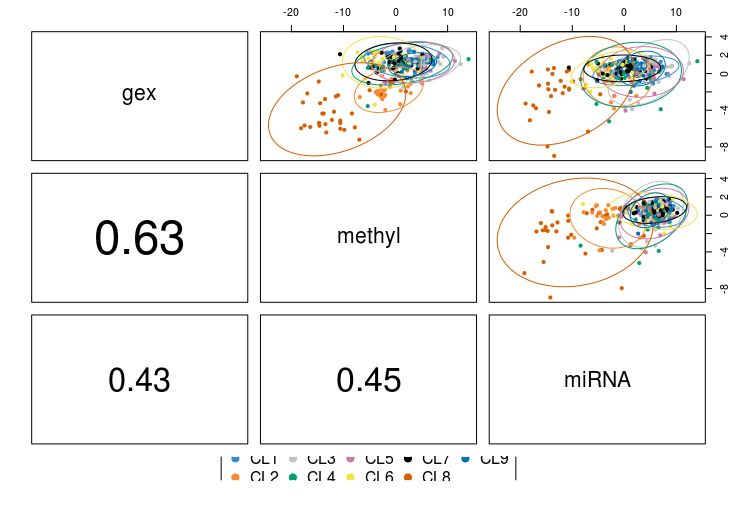 | 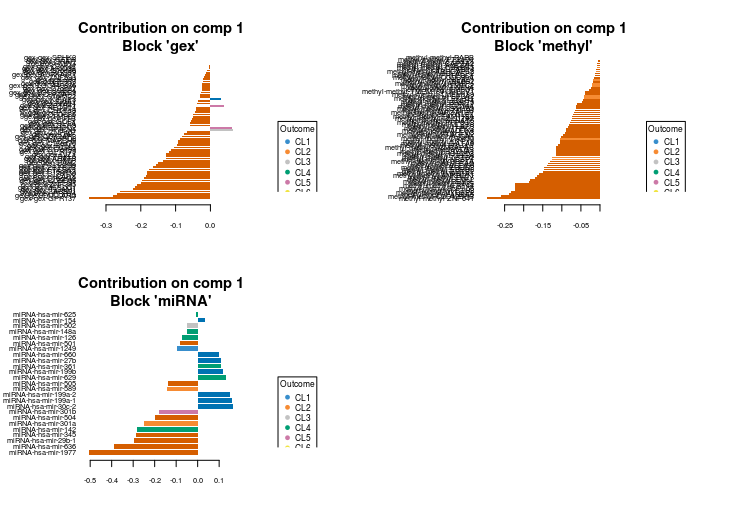 |
| --- | --- |

Component 2

| 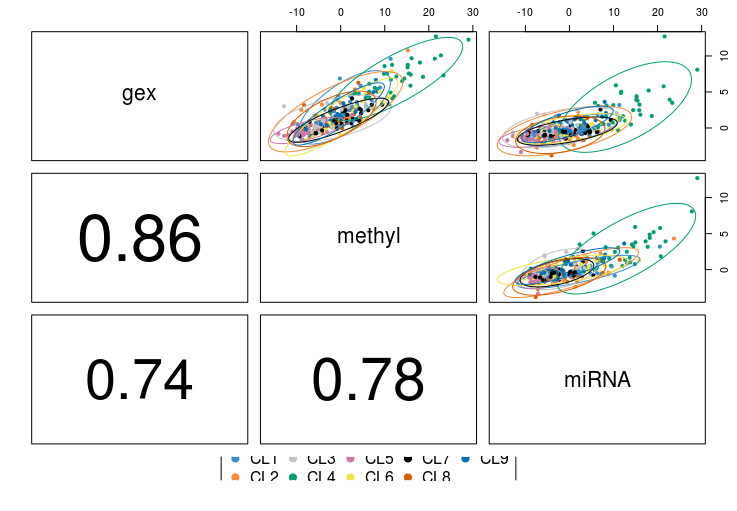 | 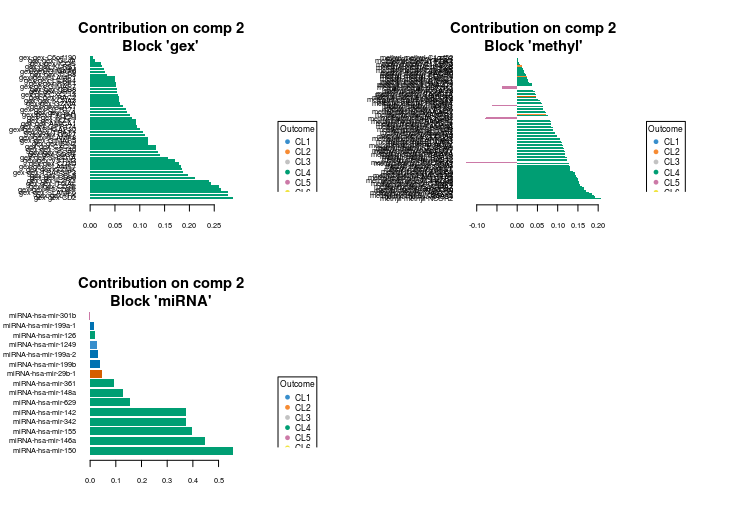 |
| --- | --- |

Component 3

| 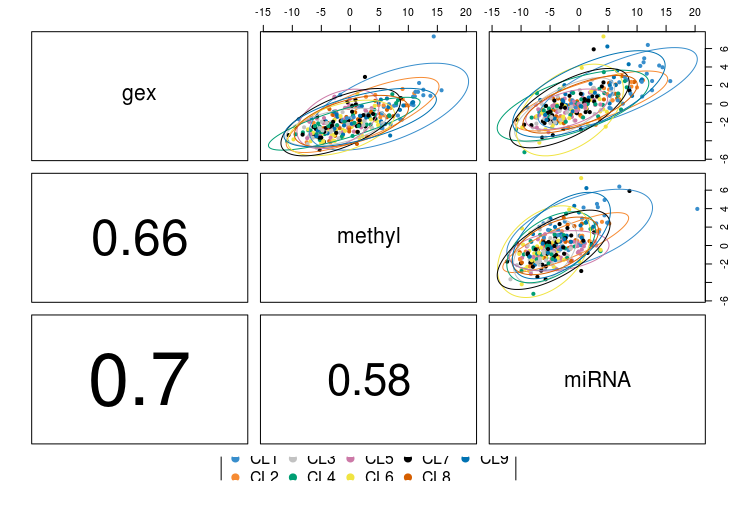 | 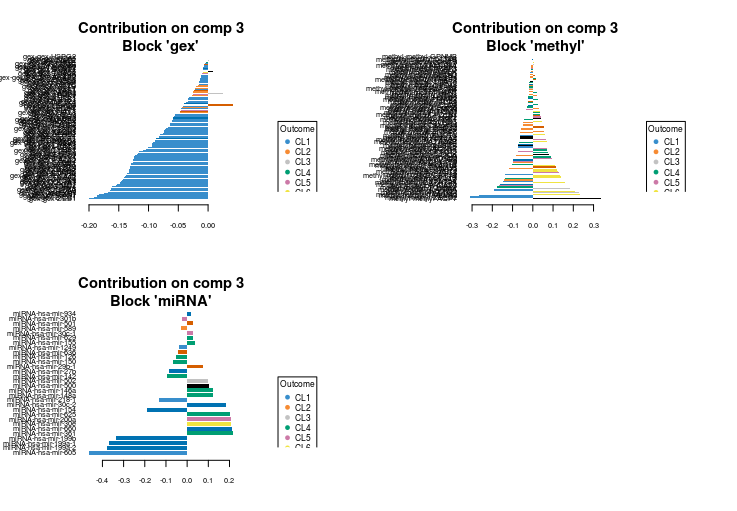 |
| --- | --- |

Component 4

| 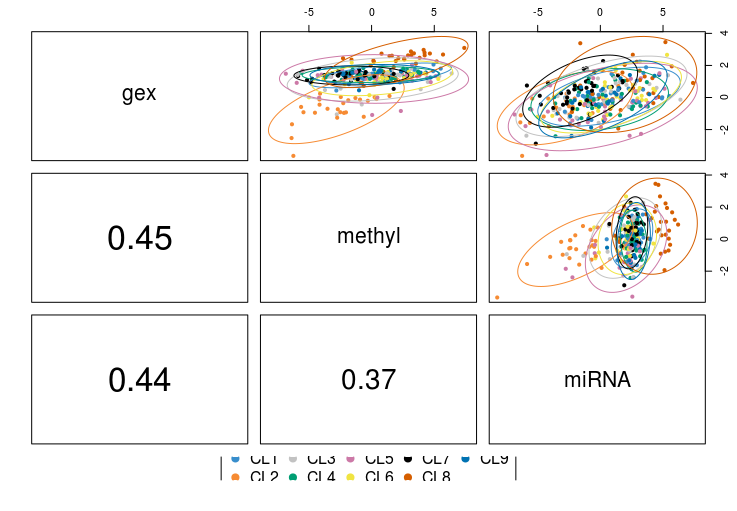 | 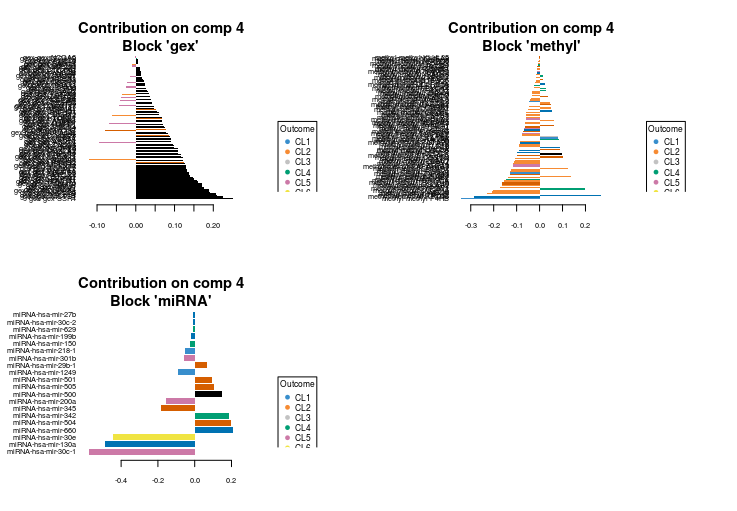 |
| --- | --- |

Component 5

| 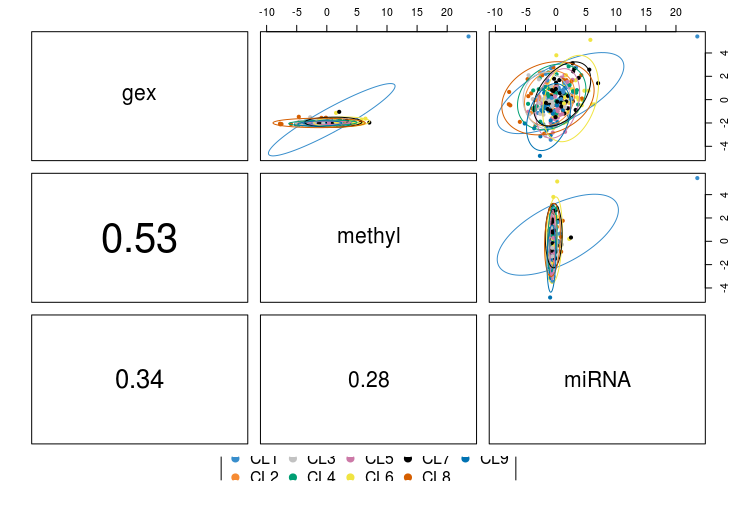 | 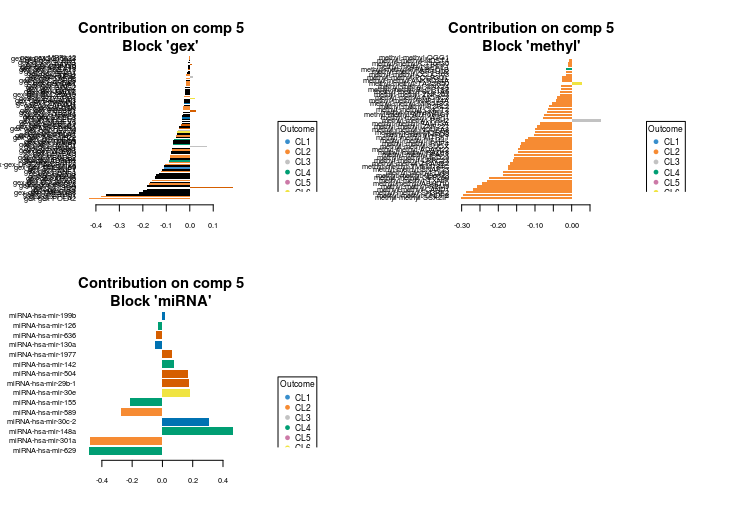 |
| --- | --- |

Component 6

| 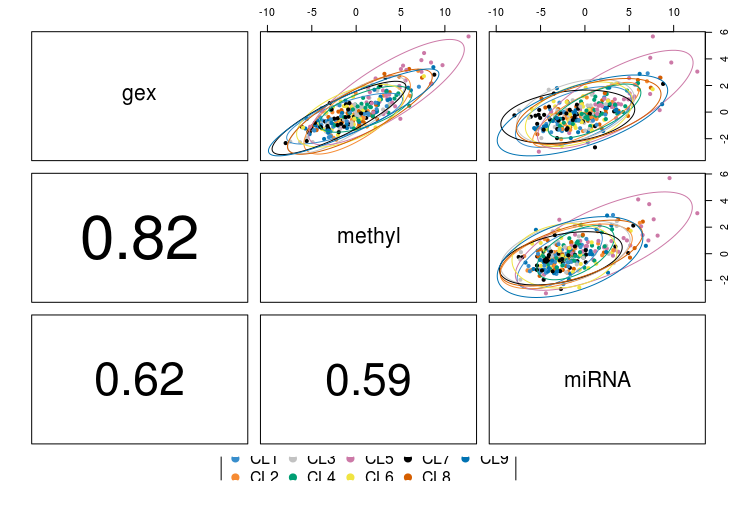 | 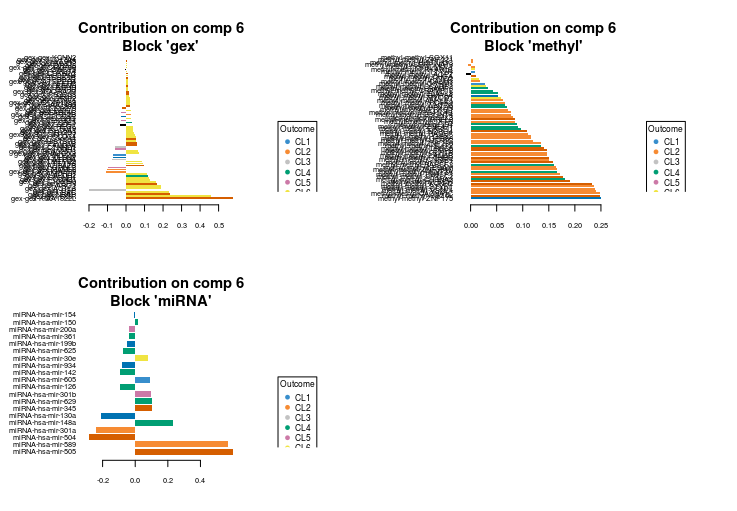 |
| --- | --- |

Component 7

| 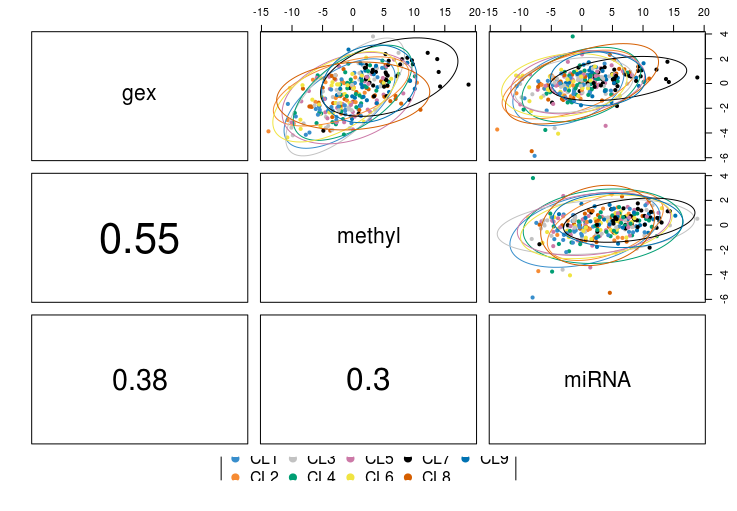 | 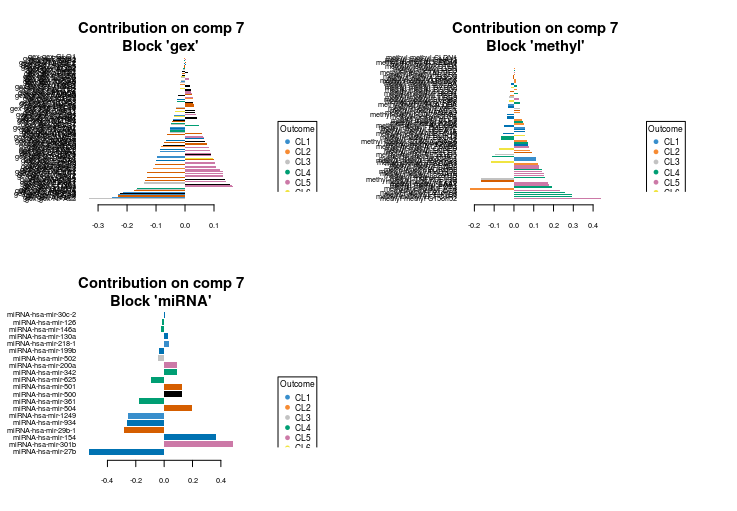 |
| --- | --- |

Component 8

| 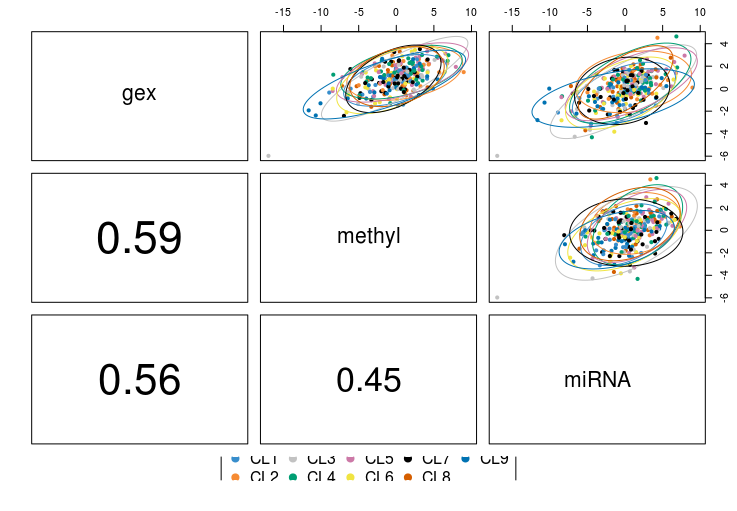 | 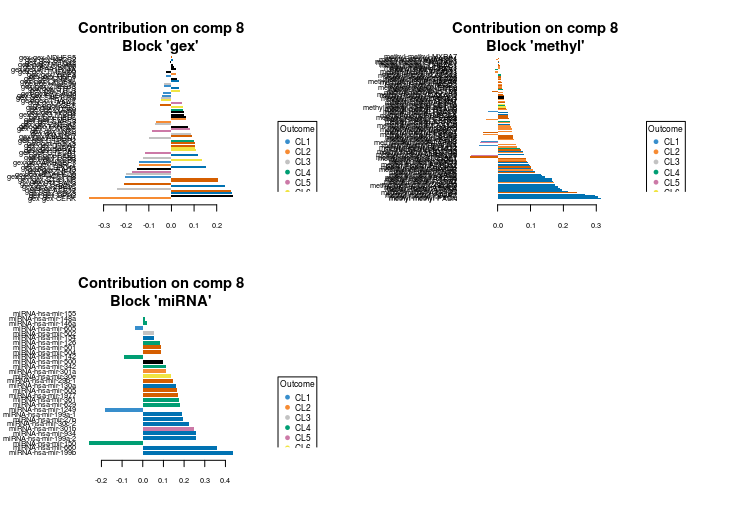 |
| --- | --- |

An overview of the clusters and the features separating them is presented in this heatmap.

The correlations between the features are presented in the circle plot below.


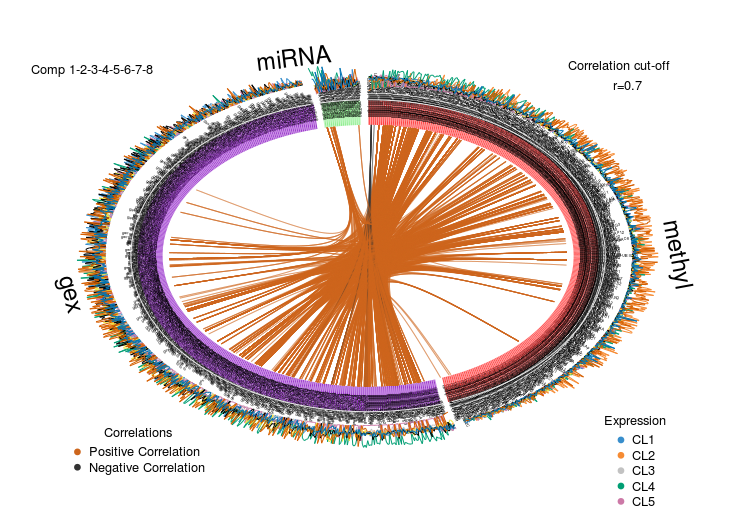


## Testing the model

The model was then tested on the testing set of data. The results are shown in the confusion matrix below.

|  | predicted.as.CL1 | predicted.as.CL2 | predicted.as.CL3 | predicted.as.CL4 | predicted.as.CL5 | predicted.as.CL6 | predicted.as.CL7 | predicted.as.CL8 | predicted.as.CL9 |
| --- | --- | --- | --- | --- | --- | --- | --- | --- | --- |
| CL1 | 0 | 0 | 0 | 10 | 7 | 0 | 0 | 0 | 0 |
| CL2 | 0 | 0 | 0 | 0 | 4 | 0 | 0 | 8 | 0 |
| CL3 | 0 | 0 | 0 | 6 | 8 | 0 | 0 | 4 | 0 |
| CL4 | 0 | 0 | 0 | 18 | 0 | 0 | 0 | 0 | 0 |
| CL5 | 0 | 0 | 0 | 0 | 17 | 0 | 0 | 2 | 0 |
| CL6 | 0 | 0 | 0 | 3 | 6 | 0 | 1 | 7 | 0 |
| CL7 | 0 | 0 | 0 | 5 | 11 | 0 | 1 | 2 | 0 |
| CL8 | 0 | 0 | 0 | 1 | 0 | 0 | 0 | 15 | 0 |
| CL9 | 0 | 0 | 0 | 6 | 7 | 0 | 0 | 0 | 0 |

The balanced error rate (BER) across all groups is of 0.68%.
